# Testing bidirectional associations of major depressive disorder with medical conditions: two-sample Mendelian randomization study

**DOI:** 10.1038/s44184-026-00204-7

**Published:** 2026-04-09

**Authors:** Yu Fang, Srijan Sen, Gita A. Pathak, Margit Burmeister, Leah S. Richmond-Rakerd

**Affiliations:** 1 Michigan Neuroscience Institute, University of Michigan, Ann Arbor, MI USA; 2 Department of Psychiatry, University of Michigan, Ann Arbor, MI USA; 3 Eisenberg Family Depression Center, University of Michigan, Ann Arbor, MI USA; 4 Institute for Genomic Health, Icahn School of Medicine at Mount Sinai, New York, NY USA; 5 Department of Genetics and Genomic Sciences, Icahn School of Medicine at Mount Sinai, New York, NY USA; 6 Department of Computational Medicine and Bioinformatics, University of Michigan, Ann Arbor, MI USA; 7 Department of Human Genetics, University of Michigan, Ann Arbor, MI USA; 8 Department of Psychology, University of Michigan, Ann Arbor, MI USA

**Keywords:** Diseases, Genetics, Medical research, Risk factors

## Abstract

Depression is associated with increased risk for a variety of medical conditions. However, the extent to which these associations reflect a causal impact of depression on medical conditions, or vice-versa, remains unresolved. We tested bidirectional causal relationships between major depressive disorder (MDD) and multiple medical conditions and symptoms, using a genetically-informed approach for causal inference. Candidate disease traits were selected based on their genetic associations with MDD, as identified in prior phenome-wide association studies that used polygenic scores for MDD and electronic health records for trait ascertainment. In total, 183 candidate traits across 15 phenome-wide association study code (phecode) categories were identified. We conducted bidirectional, two-sample Mendelian randomization using summary statistics from non-overlapping, European-ancestry genome-wide association studies (GWASs) of MDD and the disease traits. There were sufficient instrumental genetic variables to test causal effects of MDD on 182 of these traits. Genetic liability to MDD was associated with 109 (59.9%) traits, with the strongest potential causal evidence observed for 105 (57.7%) traits across 13 phecode categories: Mental disorders; digestive, genitourinary, neurological, respiratory, circulatory-system, endocrine/metabolic, musculoskeletal, sense-organ, infectious-disease, and dermatologic conditions; injuries and poisonings; and symptoms. There were 10 disease traits with sufficient instrumental genetic variables to test causal effects on MDD. Of these 10 traits, only two (20.0%)—genetically-predicted gastroesophageal reflux disease (GERD) and hypertension—were associated with MDD risk. GERD showed evidence of bidirectional associations with MDD (MDD → GERD: odds ratio (OR) = 2.02, 99% confidence interval [CI] 1.84–2.22; GERD → MDD: OR = 1.48, 99% CI 1.39–1.58). The present results are consistent with a causal effect of major depressive disorder on a broad range of medical conditions and symptoms. Prevention and treatment of MDD could benefit not only mental health but also physical health.

## Introduction

Depression is associated with a broad set of medical conditions^1^. These include diabetes and other endocrine diseases^2,3^, cardiovascular diseases^2,4^, cancer^5^, chronic respiratory diseases^4,6^, and musculoskeletal diseases^6^, among other medical conditions. Potential non-causal mechanisms for these associations include shared risk factors such as socioeconomic deprivation^7–9^ and other adversities^10,11^. Depression may also causally increase risk for medical conditions through inflammation^12,13^ and poor health behaviors^14,15^. Alternatively, medical conditions may cause depression through stress or other mechanisms^16,17^.

Determining the extent to which associations of depression with medical conditions reflect causal mechanisms is important for both psychiatric and medical interventions. If depression is a causal risk factor for medical conditions, then the prevention and treatment of depression might reduce the burden associated with medical conditions (and vice-versa). Such associations would also support the measurement of physical-health outcomes in clinical trials of mental-health treatments, and mental-health outcomes in trials of medical treatments. Further, they would inform integrated care models that coordinate medical with mental-health services^18,19^.

Mendelian randomization (MR) leverages genetic differences between people as a natural experiment to test whether an exposure confers direct risk for an outcome^20^ and represents a promising approach to understand the causal relationships between depression and medical conditions. Prior studies have used MR to interrogate associations of depression with medical conditions; for instance, diabetes^21,22^, cancers^23–25^, asthma^26^, cardiovascular diseases^27^, and gastrointestinal diseases^28^. However, most have considered only one or few conditions, and studies have not consistently tested bidirectional associations^23,24,28^. A notable exception is a study that applied MR to test bidirectional associations between major depressive disorder (MDD) and 877 disease phenotypes from the FinnGen database^29^. Results indicated that MDD may elevate risk for a range of psychiatric and systemic diseases. However, the evidence for a causal impact of other diseases on MDD was less pronounced^29^. This study provided important information about the potential diversity of diseases causally linked to MDD. However, the disease traits and associated genome-wide association study (GWAS) data were limited to the FinnGen database. Additionally, the range of sensitivity tests and negative control analyses the authors were able to conduct was constrained, limiting confidence in the robustness of the findings and interpretation of the results. The study also used summary statistics from a previous Psychiatric Genomics Consortium (PGC) GWAS meta-analysis of depression^30^ to derive instrumental variables (IVs) for MDD. A new, larger-scale PGC GWAS meta-analysis that almost doubled the number of identified loci associated with major depression is now available^31^. This provides the opportunity to ascertain more genetic instruments for MDD, increasing the power to detect causal associations of MDD with other diseases.

Phenome-wide association studies (PheWASs) can identify genetic associations between depression and other disease traits, but are limited in their ability to determine causal relationships. At the inception of this analysis (January 2024), there were six published PheWASs using polygenic risk scores for major depressive disorder (MDD-PRS) and electronic health records (EHRs) for trait ascertainment (MDD-PRS EHR PheWASs)^32–37^. Of these studies, only one followed up MDD-PRS associations using Mendelian randomization^32^. Their MR analyses indicated that MDD may be a causal risk factor for lipid-metabolism disorders, digestive-system inflammatory conditions, respiratory inflammatory conditions, and urinary-system symptoms and disorders. However, tests were limited to the disease traits associated with the MDD-PRS in that particular study, and causal effects of other traits on MDD were not tested. In the current study, we included all disease traits significantly associated with the MDD-PRS in any of these MDD-PRS EHR PheWASs as candidate traits in a bidirectional Mendelian randomization analysis. This enabled us to test potential causal associations of major depressive disorder with a broad range of associated psychiatric and medical disease traits. In addition to utilizing a PheWAS-informed disease selection approach and implementing bidirectional tests, the current study also expanded on prior work by using the most up-to-date GWAS meta-analysis of major depression^31^ to derive genetic instruments for MDD.

## Methods

### Trait selection and GWAS dataset search

We used PheWAS codes (phecodes) to select candidate disease traits. Phecodes are manually-curated groups of International Classification of Diseases (ICD) codes designed to represent clinically-significant concepts for research purposes^38^. We selected 249 phecode traits previously found to be significantly associated with the MDD-PRS in published MDD-PRS PheWASs^32–37^. Three traits (major depressive disorder [phecode 296.22], depression [phecode 296.2], and mood disorders [phecode 296]) were excluded due to their inclusion of MDD, leaving 246 candidate traits (Supplementary Data 1).

**Fig. 1:**
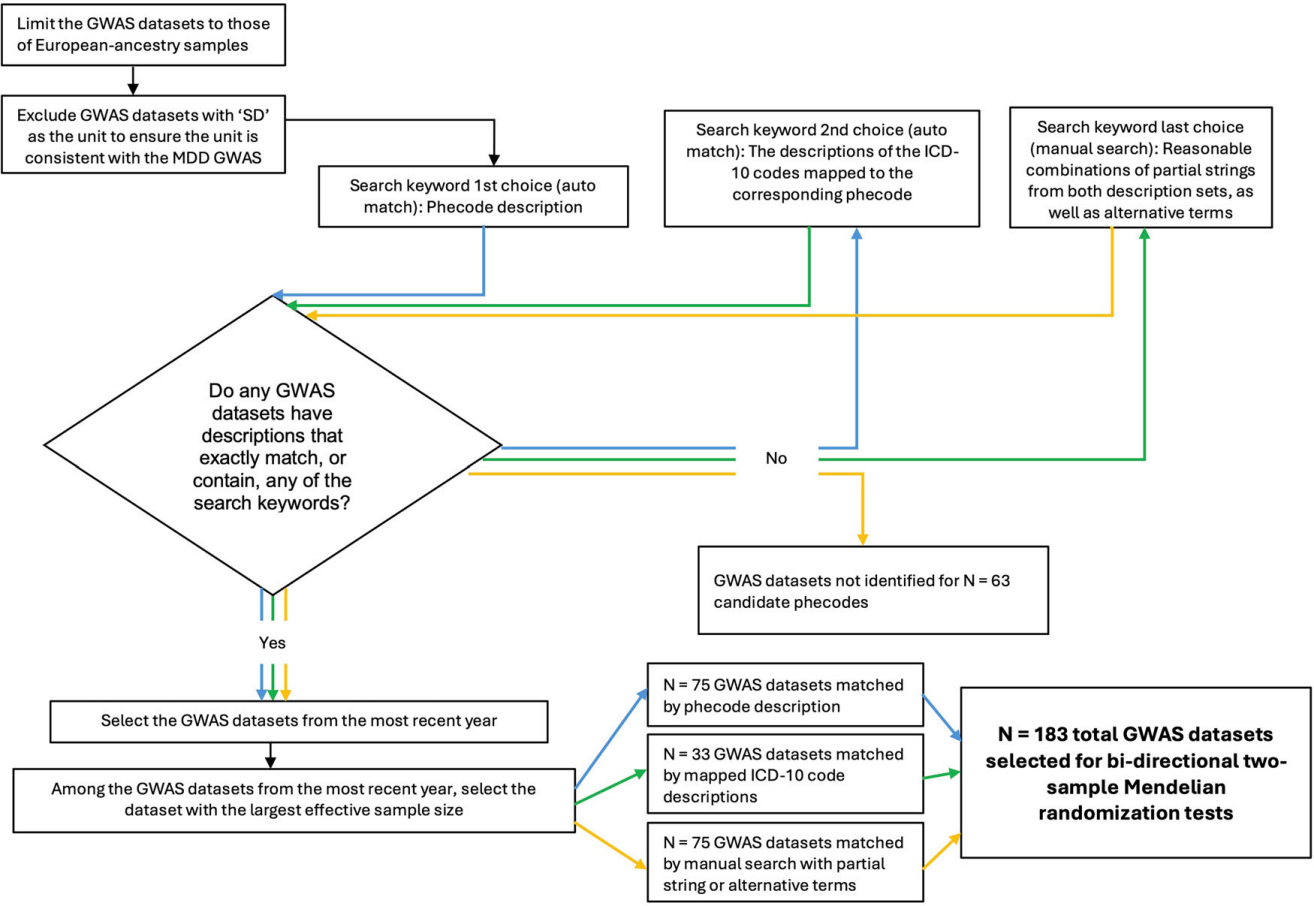
GWAS dataset search pipeline.

Subsequently, we searched GWAS datasets corresponding to the 246 candidate traits in the MRC Integrative Epidemiology Unit (IEU) GWAS database^39,40^. We limited the GWAS datasets to European-ancestry samples. We also excluded GWAS datasets with ‘SD’ as the unit to ensure that the unit was consistent with the MDD GWAS. For each phecode, we first used the phecode trait description as the search keyword to identify the GWAS dataset. If none were found using the phecode trait description, we searched using the descriptions of the corresponding ICD-10 codes, using Phecode Map 1.2^41^. If none were found using the ICD-10 code descriptions, we manually tested reasonable combinations of partial strings from both description sets, as well as alternative terms. When multiple GWAS datasets were identified, we selected the dataset from the most recent year, and of those, the dataset with the largest effective sample size. The details of the GWAS search pipeline are described in Fig. 1. Supplementary Data 2 shows the candidate and final matching keywords for each phecode. A total of 183 GWAS datasets of European-ancestry samples representing the candidate traits were identified in the IEU GWAS database and utilized in the two-sample MR tests (Supplementary Data 1).

The GWAS datasets available in the IEU GWAS database comprise a substantial number of studies derived from UK Biobank samples. To minimize the risk of sample overlap and reduce bias in effect size estimation^42^, we used the MDD GWAS summary statistics from the meta-analysis of PGC MDD Phase 3, excluding 23andMe and UK Biobank samples, containing 357,636 cases and 1,281,936 controls of European ancestry^31^.

### Two-sample Mendelian randomization

To test potential causal relationships between MDD and the traits that showed significant associations with the MDD-PRS in prior EHR PheWASs, we employed bidirectional, two-sample Mendelian randomization. Our tests followed established protocols and incorporated evaluation of potential violations of MR assumptions^20,30,43^. MR analyses assume that the instrumental genetic variables (a) are strongly associated with the exposure, (b) are independent of confounders of the exposure-outcome relationship, and (c) affect the outcome only through the exposure^44^.

First, to obtain the instrumental genetic variables for the two-sample MR analysis, we extracted the single-nucleotide polymorphisms (SNPs) that reached genome-wide significance (*p* < 5 × 10^−8^) in the exposure GWAS dataset for each MDD-disease set. We selected genome-wide significant SNPs to help ensure that the genetic instruments were strongly associated with the exposure and reduce the potential for weak-instrument bias. We then clumped the identified SNPs with a 10,000 kb window and a cutoff of r^2^ < 0.001. Next, the same SNPs or their proxy SNPs (r^2^ > 0.8) were extracted from the outcome GWAS dataset. Finally, the effects of the selected SNPs on the exposure and outcome datasets were harmonized to be relative to the same alleles.

We implemented a multi-step approach to test each MDD-disease set:

1) We restricted our test sets for analysis to those with sufficient instrumental-variable SNPs (n_variable_ > =30)^30^.

2) We performed the MR Egger intercept test to assess for association due to directional horizontal pleiotropy. β_intercept_ with a significance level of *p* < 0.05 would suggest that the genetic instruments impact the outcome through pathways other than the exposure, thereby violating the exclusion restriction assumption^45^.

3) To assess causal relationships, we used the inverse-variance weighted (IVW) test, and calculated the false discovery rate (FDR)-adjusted p value and q value. An FDR-adjusted *p* < 0.01 would indicate a potential causal relationship.

4) For the test sets that passed the IVW test, we conducted a variant heterogeneity test with IVW regression.

5) Any test sets with a significance level of IVW *p* < 0.05 in the variant heterogeneity test underwent additional sensitivity tests, including the weighted median test and MR Egger test. These sensitivity tests were considered successful if their effects were in the same direction as the IVW test.

6) We applied a series of ‘leave-one-variant-out’ IVW tests to evaluate whether the observed effect was driven by a single variant (*p*_max_<0.05).

7) For the MDD-disease sets that passed the above tests, we conducted three additional sets of analyses. First, the MR-PRESSO test was used to identify outlier IVs with horizontal pleiotropy and perform outlier-removed MR analysis^46^. Second, the MR-Horse test was applied to correct for correlated pleiotropic effects^47^. Third, Steiger filtering was implemented to detect reverse-causal IVs and carry out reverse-causal-IV-removed MR analysis^48,49^.

Overall, the test sets that had no evidence of directional horizontal pleiotropy and had an FDR-adjusted *p* < 0.01 in the IVW test were considered to have evidence of a putative causal effect. In cases where the test sets exhibited no signs of variant heterogeneity or successfully passed the additional sensitivity tests, and the observed effect was not solely influenced by a single variant, then the causal effect was regarded as relatively robust. Evidence for the causal effect was further strengthened if MR results remained significant after the removal of outlier IVs exhibiting horizontal pleiotropy or reverse causality, and after correction for correlated pleiotropic effects. Supplementary Fig. 1 depicts the pipeline.

After all test sets were examined, we calculated the proportion of test sets that exhibited evidence of causality in the direction of MDD to other disease traits, and other traits to MDD.

The power of two-sample MR tests to detect causal effects increases with larger sample sizes of exposure and outcome GWAS datasets^50^. To reduce the bias arising from variations in sample size across the GWAS datasets for different traits, we repeated the proportion calculation, considering only the test sets with exposure and outcome datasets with an effective sample size >=100,000 or number of cases >=10,000.

In previous two-sample MR analyses^29,30,51,52^, various thresholds for the minimum number of IVs to include have been utilized. Thus, we performed a sensitivity analysis in which the threshold for the minimum number of instrumental-variable SNPs was relaxed from 30 to 5.

As an additional test for the influence of GWAS dataset sample size on discovery of causal effects, we used a GWAS dataset of height including three million European-ancestry individuals^53^ as a negative control. This enabled us to examine whether a very large GWAS dataset not expected to be causally related to many MDD-PRS-associated traits showed spurious evidence of causal effects on these traits because of high power.

### Instrumental variables strength

To further evaluate the potential for weak-instrument bias, we used the F-statistic (*F* = beta^2^/SE^2^) to estimate the strength of IVs in the MR tests^54,55^, where beta and SE represent the effect size and standard error, respectively, of the genetic variant on the risk of exposure. We examined the F-statistic for each genetic variant and calculated the mean F-statistic for each MDD-disease set. Any genetic variant with an F-statistic <10 would be considered to have a poor association with the exposure trait and weak-instrument bias^20^.

Analyses were conducted using the R package ‘TwoSampleMR’ v0.6.9^40,48^.

## Results

Of the 183 GWAS datasets for which candidate traits were identified, there were sufficient IVs (n_variable_ > =30) to test causal effects of MDD on 182 other disease traits, and 10 other disease traits on MDD. The mean instrument F-statistics exceeded conventional thresholds (>=10) across analyses, ranging from 29.6 to 1691.3 for individual instrument variables and from 38.7 to 110.8 for the mean of each test.

**Fig. 2:**
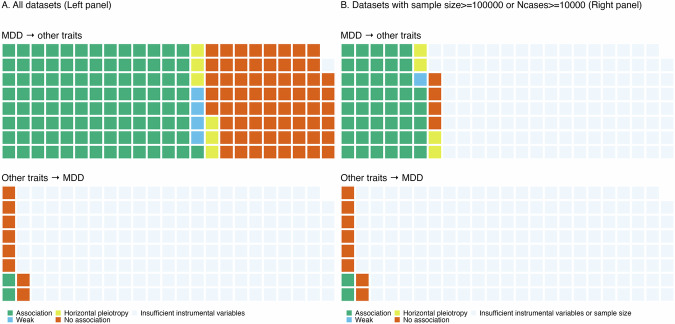
Summary of two-sample Mendelian randomization tests between major depressive disorder and MDD-PRS-associated traits. Association: Tests reached the 1% significance threshold after FDR correction in the IVW analysis; had no evidence of variant heterogeneity, or had effects in the same direction in the sensitivity tests when having evidence of variant heterogeneity; and results remained consistent in the ‘leave one variant out’ IVW analysis. Weak: Tests reached the 1% significance threshold after FDR correction in the IVW analysis but had evidence of variant heterogeneity, and did not have effects in the same direction in the sensitivity tests. Horizontal pleiotropy: Tests showed evidence of directional horizontal pleiotropy in the MR Egger test, indicating that the genetic instruments impacted the outcome through biological pathways other than the exposure. No association: Tests did not reach the 1% significance threshold after FDR correction in the IVW analysis. Insufficient instrumental variables: Number of instrumental variables<30. Insufficient sample size: Datasets with sample size < 100,000 and Ncases < 10,000.

We identified putative causal effects of MDD on 109/182 other disease traits (59.9% of total tests) at a 1% significance threshold after FDR correction in IVW regression analysis. All of these associations were positive, indicating that the presence of MDD causally increased the risk of the disease traits (Fig. 2A upper, Supplementary Fig. 2, Supplementary Data 3). Of the 109 putative causal associations, four had evidence of variant heterogeneity, and weighted median and MR Egger tests yielded results in the opposite direction of effect as the IVW test. The 105 remaining associated traits (57.7% of total tests) for which there was stronger evidence for a causal effect of MDD remained significant after the removal of outlier IVs exhibiting horizontal pleiotropy (MR-PRESSO) and/or reverse causality (Steiger filtering), and also after correction for correlated pleiotropic effects (MR-Horse). Of these 105 traits, 97 reached *p* < 0.05 and 87 reached *p* < 0.01 in weighted median tests (Supplementary Data 4).

The 105 traits with stronger evidence for a causal effect of MDD belonged to a broad range of phecode categories (13 of the 15 categories included in analyses [Table 1A, Supplementary Data 3]). These categories were mental disorders; digestive, genitourinary, neurological, respiratory, circulatory-system, endocrine/metabolic, musculoskeletal, sense-organ, infectious-disease, and dermatologic conditions; injuries and poisonings; and symptoms. The proportion calculation was similar when traits in the injuries and poisonings and symptoms categories were excluded (93/167; 55.7%).

**Table 1:** Top 5 significant associations for tests of A) MDD’s causal effects on other traits and B) other traits’ causal effects on MDD, per phecode group

Phecode Group	Phecode	Phecode Description	MR IVW test			
A. Top Associations for Tests of MDD’s Causal Effects on Other Traits						
			beta	OR	OR 99%CI	FDR *q*-value
Infectious Diseases	112	candidiasis	0.44	1.56	1.12–2.16	2.29E-04
	41	bacterial infection nos	0.12	1.13	1.01–1.26	1.98E-03
Endocrine/metabolic	278	overweight, obesity and other hyperalimentation	0.47	1.60	1.30–1.98	9.03E-09
	278.1	obesity	0.47	1.60	1.29–1.97	1.22E-08
	250.2	type 2 diabetes	0.25	1.28	1.11–1.48	5.91E-06
	250	diabetes mellitus	0.20	1.22	1.07–1.40	4.81E-05
	276	disorders of fluid, electrolyte, and acid-base balance	0.35	1.42	1.12–1.79	5.69E-05
Mental Disorders	306.1	mental disorders durring/after pregnancy	0.70	2.01	1.83–2.22	7.15E-76
	297.2	suicide or self-inflicted injury	0.70	2.02	1.83–2.23	2.08E-74
	300	anxiety disorders	0.77	2.17	1.90–2.47	4.80E-51
	303	psychogenic and somatoform disorders	0.77	2.16	1.90–2.47	3.01E-50
	300.1	anxiety disorder	0.94	2.56	2.11–3.10	8.70E-35
Neurological	338	pain	0.43	1.53	1.40–1.68	3.95E-32
	353	nerve root and plexus disorders	0.36	1.43	1.26–1.63	2.19E-12
	339	other headache syndromes	0.54	1.72	1.40–2.11	1.64E-11
	327.3	sleep apnea	0.42	1.52	1.29–1.78	6.04E-11
	327	sleep disorders	0.35	1.42	1.22–1.64	8.00E-10
Sense organs	386.9	dizziness and giddiness (light-headedness and vertigo)	0.31	1.36	1.18–1.57	1.26E-08
	368	visual disturbances	0.25	1.28	1.07–1.53	1.52E-04
	386.2	peripheral or central vertigo	0.22	1.25	1.04–1.49	5.72E-04
Circulatory system	418	nonspecific chest pain	0.39	1.48	1.35–1.62	4.22E-27
	411.1	unstable angina (intermediate coronary syndrome)	0.38	1.46	1.24–1.71	2.58E-09
	411.3	angina pectoris	0.32	1.38	1.17–1.64	6.06E-07
	433.31	transient cerebral ischemia	0.36	1.43	1.18–1.73	9.67E-07
	428.2	heart failure nos	0.16	1.17	1.06–1.30	2.87E-05
Respiratory	496.2	chronic bronchitis	0.64	1.89	1.57–2.27	3.51E-18
	513	respiratory abnormalities	0.38	1.46	1.31–1.63	3.80E-18
	496	chronic airway obstruction	0.50	1.65	1.42–1.93	1.41E-16
	495	asthma	0.25	1.28	1.16–1.41	2.85E-11
	497	bronchitis	0.26	1.30	1.17–1.45	2.24E-10
Digestive	530.11	gerd	0.70	2.02	1.84–2.22	5.88E-82
	561	symptoms involving digestive system	0.41	1.51	1.37–1.66	2.17E-28
	564.1	irritable bowel syndrome	0.56	1.76	1.41–2.19	6.86E-11
	526.42	arthralgia/ankylosis of temporomandibular joint	0.75	2.12	1.57–2.86	1.64E-10
	564	functional digestive disorders	0.45	1.56	1.30–1.88	4.02E-10
Genitourinary	626.1	irregular menstrual cycle/bleeding	0.41	1.51	1.30–1.75	6.77E-13
	591	urinary tract infection	0.36	1.43	1.25–1.63	8.03E-12
	599	other symptoms/disorders or the urinary system	0.30	1.35	1.20–1.53	2.16E-10
	628	ovarian cyst	0.37	1.45	1.24–1.70	9.93E-10
	592	cystitis and urethritis	0.36	1.43	1.19–1.72	2.94E-07
Dermatologic	681	superficial cellulitis and abscess	0.29	1.33	1.15–1.55	3.72E-07
	687.4	disturbance of skin sensation	0.43	1.54	1.20–1.98	5.95E-06
Musculoskeletal	724	other and unspecified disorders of back	0.45	1.57	1.37–1.79	1.17E-17
	722.9	other and unspecified disc disorder	0.44	1.56	1.33–1.82	3.17E-13
	745	pain in joint	0.44	1.55	1.32–1.82	2.25E-12
	721	spondylosis and allied disorders	0.46	1.58	1.30–1.91	9.28E-10
	740	osteoarthrosis	0.24	1.27	1.14–1.43	1.66E-08
Symptoms	760	back pain	0.53	1.70	1.53–1.90	2.58E-37
	785	abdominal pain	0.43	1.54	1.40–1.70	1.05E-29
	765	cervical radiculitis	0.46	1.58	1.31–1.92	5.76E-10
	764	sciatica	0.48	1.61	1.32–1.97	1.21E-09
	761	cervicalgia	0.61	1.84	1.41–2.39	3.14E-09
Injuries & Poisonings	961	poisoning by other anti-infectives	0.96	2.61	2.01–3.37	4.04E-21
B. Top Associations for Tests of Other Traits’ Causal Effects on MDD						
Circulatory system	401	hypertension	0.03	1.03	1.01–1.06	6.39E-03
Digestive	530.11	gerd	0.39	1.48	1.39–1.58	3.25E-53

Traits are ranked by IVW test FDR q-values; if fewer than five significant causal associations exist within a group, then all the causal associations within that group are listed

To identify top associations, we selected the traits with an FDR-adjusted *p* < 1 × 10^−10^ in the IVW test. For these 29 traits, the maximum weighted median test *p*-value was smaller than 1 × 10^−4^, and 21 of the GWAS datasets had an effective sample size of at least 100,000 participants or 10,000 cases (Supplementary Fig. 3, Supplementary Data 4). These 29 traits included:

12 traits in the mental disorders phecode category: mental disorders during/after pregnancy, suicide or self-inflicted injury, anxiety disorders [and its sub-trait, anxiety disorder], psychogenic and somatoform disorders, neurological disorders, personality disorders, adjustment reaction, substance addiction and disorders, bipolar disorder, alcohol-related disorders, and generalized anxiety disorder (effect size range: beta = 0.37–0.99 and odds ratio [OR] = 1.45–2.68 [IVW test], beta = 0.32–0.99 and OR = 1.38–2.69 [weighted median test]);

Six pain-related traits: back pain, pain, abdominal pain, nonspecific chest pain, pain in joint, and other headache syndromes (effect size range: beta = 0.39–0.54 and OR = 1.48–1.72 [IVW test], beta = 0.35–0.54 and OR = 1.41–1.71 [weighted median test]);

Three traits in the respiratory category: chronic bronchitis, respiratory abnormalities, and chronic airway obstruction (effect size range: beta = 0.38–0.64 and OR = 1.46–1.89 [IVW test], beta = 0.38–0.58 and OR = 1.47–1.78 [weighted median test]);

Two traits in the digestive category: gastroesophageal reflux disease (GERD) and digestive system symptoms (effect size range: beta = 0.41–0.70 and OR = 1.51–2.02 [IVW test], beta = 0.38–0.66 and OR = 1.46–1.93 [weighted median test]);

Two traits in the musculoskeletal category: other and unspecified disorders of back and other and unspecified disc disorder (effect size range: beta = 0.44–0.45 and OR = 1.56–1.57 [IVW test], beta=0.37–0.46 and OR = 1.44–1.59 [weighted median test]);

Two traits in the genitourinary category: irregular menstrual cycle/bleeding and urinary tract infection (effect size range: beta = 0.36–0.41 and OR = 1.43–1.51 [IVW test], beta = 0.40–0.44 and OR = 1.49–1.56 [weighted median test]);

One trait from the neurological category: nerve root and plexus disorders (effect size: beta = 0.36 and OR = 1.43 [IVW test], beta = 0.28 and OR = 1.32 [weighted median test]);

One trait from the injuries and poisonings category: poisoning by other anti-infectives (effect size: beta = 0.96 and OR = 2.61 [IVW test], beta = 0.88 and OR = 2.42 [weighted median test]).

When considering causal effects of other disease traits on MDD, positive putative causal effects were identified for 2/10 traits (20.0%): GERD (beta = 0.39, OR = 1.48 [99% confidence interval {CI}: 1.39–1.58], *p*_FDR_ = 3.62 × 10^−53^ [IVW test]; beta=0.31, OR = 1.36 [99%CI: 1.28–1.44], *p* = 2.65 × 10^−39^ [weighted median test]) and hypertension (beta=0.03, OR = 1.03 [99%CI: 1.01–1.06], *p*_FDR_ = 0.007 [IVW test]; beta=0.03, OR = 1.03 [99%CI: 1.01–1.06], *p* = 0.002 [weighted median test]) (Fig. 2A lower). Both associations remained significant after the removal of outlier IVs exhibiting horizontal pleiotropy (MR-PRESSO) and/or reverse causality (Steiger filtering), and after correction for correlated pleiotropic effects (MR-Horse) (Supplementary Data 5 and Supplementary Fig. 4BA and BH).

**Fig. 3:**
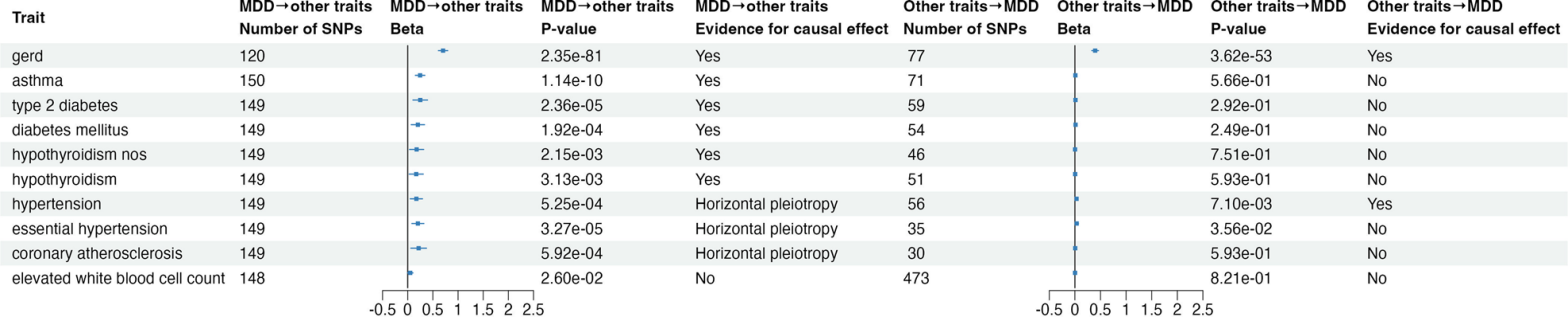
Results of bidirectional Mendelian randomization analyses of major depressive disorder and 10 MDD-PRS-associated traits, conducted using the IVW method. Causal-effect estimates are presented as beta coefficients with 99% confidence intervals. All traits are binary, with the exception of “elevated white blood cell count” (continuous). Odds ratios for associations with binary traits are shown in Supplementary Data 4 and 5.

For the 10 traits for which causal relationships with MDD were tested in both directions, six (60.0%; GERD, asthma, type 2 diabetes, diabetes mellitus, hypothyroidism NOS, and hypothyroidism) showed evidence of being caused by MDD, while two (20.0%; GERD and hypertension) showed evidence of causal effects on MDD (Fig. 3, Supplementary Fig. 4). GERD was the only trait that showed evidence of causal effects in both directions (MDD → GERD: IVW beta=0.70, OR = 2.02 [99%CI: 1.84–2.22], *p*_FDR_ = 1.29 × 10^−83^; GERD → MDD: IVW beta=0.39, OR = 1.48 [99%CI: 1.39–1.58], *p*_FDR_ = 3.62 × 10^−53^). The associations remained significant in the weighted median test, after the removal of outlier IVs exhibiting horizontal pleiotropy (MR-PRESSO) and/or reverse causality (Steiger filtering), and after correction for correlated pleiotropic effects (MR Horse; Table 1B, Supplementary Data 4 and 5).

When exclusively considering test sets with exposure and outcome datasets with an effective sample size >=100,000 or >=10,000 cases, putative causal effects of MDD on 54 other traits and causal effects of 10 other traits on MDD could be considered. Putative causal effects of MDD on 46 other traits (85.2% of total tests, one with weaker evidence) were detected (Fig. 2B upper, Supplementary Data 4). The 45 traits (83.3% of total tests) with stronger causal evidence belonged to 12 phecode categories: mental disorders; digestive, genitourinary, neurological, respiratory, circulatory-system, endocrine/metabolic, musculoskeletal, sense-organ, infectious-disease, and dermatologic conditions; and symptoms. Putative causal effects of two out of 10 traits on MDD were detected (GERD and hypertension; 20.0% of total tests; Fig. 2B lower, Supplementary Data 5).

When relaxing the minimum number of IVs to n_variable_ > =5, there were sufficient IVs to test causal effects of MDD on 183 other disease traits (one more trait than in primary analyses). There were sufficient IVs to test causal effects of 41 other disease traits on depression (31 more traits than in primary analyses). We identified putative causal effects of MDD on 109/183 other disease traits (59.6% of total tests, four with weaker evidence) and 1/41 other disease traits on MDD (GERD, 2.4% of total tests).

In the series of tests using height as a negative control, only 16 putative causal effects of height on other traits were detected (8.7% of 183 total tests; Supplementary Data 6). These included both positive and negative associations. Several of these associations have been reported in prior literature, including an inverse association with hypertension^56^ and a potential positive association with osteoarthrosis^57^. These findings provide further evidence that the detected effects of MDD on other disease traits are unlikely to be a spurious result of the large MDD GWAS sample size.

## Discussion

In this bidirectional Mendelian randomization analysis using genetic instruments for major depressive disorder and MDD-PRS-associated medical conditions from the electronic health record, we identified putative causal effects of major depressive disorder on a broad range of disease traits across the clinical phenome. Among tests for disease traits causally related to MDD, only gastroesophageal reflux disease and hypertension were causally implicated in MDD risk.

Specifically, we detected putative causal effects of MDD on 109 of 182 (59.9%) traits. After removing traits with weaker causal evidence, putative effects on 105 of 182 (57.7%) traits across 13 phecode categories remained. Further, when restricting to traits from the largest-scale GWASs, putative causal effects on 46 of 54 (85.2%) traits across 12 phecode categories were detected. As expected, traits in the mental-disorders category were among the most strongly associated. However, top associations also emerged with a range of other medical conditions and symptoms. These included pain-related traits as well as traits in the respiratory, digestive, musculoskeletal, genitourinary, neurological, and injuries and poisonings categories.

Across 10 traits tested bidirectionally, there was evidence for a causal impact of MDD on risk for GERD, asthma, diabetes mellitus, Type 2 diabetes, hypothyroidism NOS, and hypothyroidism. In contrast, only two traits—GERD and hypertension—showed evidence of a causal influence on risk for MDD. The F-statistics for the instrumental genetic variables exceeded conventional thresholds across analyses, indicating that weak-instrument bias is unlikely to explain the largely null results for the bidirectional tests. Findings from prior bidirectional Mendelian randomization studies of depression and GERD are mixed; some indicate causal associations in both directions^58,59^, and others suggest that GERD increases risk for depression, but not vice-versa^60^. In contrast to our findings, previous bidirectional MR studies of hypertension did not find evidence for a causal effect of hypertension on depression^52,61^. Also in contrast, a recent bidirectional MR analysis did not find evidence for a causal effect of depression on hypothyroidism, but did observe evidence for a causal effect of hypothyroidism on depression^62^. Our findings broadly align with prior bidirectional MR studies of asthma^26^ and Type 2 diabetes^21,22^, which found that depression may increase risk for these conditions, but they may not confer risk for depression. Previous bidirectional MR data concerning diabetes mellitus or broad diabetes (combining Type 1 and Type 2) are limited, but existing MR evidence suggests that diabetes might reduce risk for depression, with no causal evidence in the reverse direction^63^. Although Type 2 diabetes and diabetes mellitus are related traits, they were analyzed separately in the current report as they have distinct phecode definitions.

When the minimum number of IVs was relaxed to five, we were able to expand our bidirectional tests to include 31 additional disease traits. Of the 41 traits evaluated, only one (GERD) had evidence of a putative causal effect on MDD, after correction for multiple testing. Thus, even with a larger number of tested traits, the proportion of disease traits with evidence for a causal effect on MDD was much lower than vice-versa. However, it should be noted that these analyses may have had reduced power and precision for detecting causal relationships due to including fewer IVs^64,65^.

It is informative to compare our results against those from two previous bidirectional Mendelian randomization studies of MDD and medical conditions. A prior MDD-PRS EHR PheWAS (from a GWAS of MDD^66^ over three times smaller than the GWAS used for the current analysis) employed Mendelian randomization to follow up MDD-PRS associations with 46 traits^32^. In both their study and ours, putative causal effects of MDD were identified for traits related to anxiety, sleep, pain, urinary-system disorders, ovarian cysts, asthma, arthritis, obesity, and pneumonia. Additionally, a large-scale analysis of major depressive disorder and 877 other disease phenotypes from the FinnGen database^29^ found evidence of a causal impact of MDD on medical diseases across diverse body systems. Evidence for a causal impact of other diseases on MDD was less marked. Here, we used a disease-ascertainment approach informed by prior MDD-PRS phenome-wide association studies^32–37^, a series of sensitivity and robustness tests, and genetic instruments for MDD drawn from the most up-to-date GWAS meta-analysis of major depression^31^. This enabled us to detect putative causal effects of MDD on traits across many of the same body systems, and also traits in additional systems, including sense-organ and dermatologic conditions. Consistent with the prior report^29^, we also found greater evidence for causal effects of MDD on other disease traits than vice-versa. Taken together, these findings increase confidence that MDD causes a substantial burden of comorbidity across diverse body systems. Additionally, the causal pathway from MDD to systemic diseases appears to be stronger than the pathway from systemic diseases to MDD.

Our study has strengths. First, we tested associations of major depressive disorder with disease traits across a range of domains, enabling us to comprehensively characterize causal disease effects. Second, we drew on multiple MDD-PRS EHR PheWASs to inform our selection of disease traits, reducing potential bias in trait ascertainment given variation in sample size and polygenic risk score construction across studies. Third, our bidirectional approach allowed us to consider reciprocal associations between MDD and medical conditions and symptoms, informing understanding of the potential health benefits of both psychiatric and medical interventions. Fourth, we employed multiple sensitivity tests and an informative negative control analysis, strengthening conclusions regarding the robustness of our findings.

We also acknowledge limitations. First, not all disease traits could be identified in the IEU GWAS database, and insufficient IVs limited the number of traits for which we could test causal effects on MDD. However, when relaxing the requirement for the minimum number of instrumental genetic variables, we tested causal effects of a larger number of disease traits on MDD and still detected very few causal effects. This suggests that a greater proportion of genetic associations of MDD with other medical conditions arise in part from a causal effect of MDD, rather than vice-versa. However, large-scale GWASs of diseases across the clinical phenome will be necessary to confirm this conclusion. This should represent a focus for future GWAS consortium efforts. Second, verification of the potential causal effects identified here may require randomized controlled trials of preventive interventions. However, some trials could be infeasible due to long follow-ups and high cost. MR, therefore, represents an important component of triangulation efforts to characterize causal associations of MDD with medical conditions. Third, Mendelian randomization estimates represent lifelong average effects of genetic variants, and are not informative about effects of a risk factor at a specific time, or an intervention of discrete duration^20^. Thus, our findings cannot speak to, for instance, the potential effects of time-limited MDD treatments delivered at particular life stages on longer-term risk for physical disease. Fourth, we did not test mediation. Thus, unquantified mediation may partly account for the associations observed here. Additionally, traits with which we identified putative causal associations with MDD may share contributing causes. As one example, poor health behaviors may increase risk for both MDD and GERD. Future work should implement MR-based mediation (e.g., multivariable MR^67^) to test mediation of associations by plausible potential mechanisms (for instance, smoking, alcohol use, and BMI) and evaluate the potential for shared confounding. Fifth, some traits belonging to different phecode categories were similar (e.g., ‘pain’ in the neurological category and ‘pain in joint’ in the musculoskeletal category). This reflects the conceptual similarity of some diseases and their multifactorial nature, but also means that the total number of ‘unique’ traits analyzed may be lower. Lastly, the exposure and outcome GWAS datasets used for MR analyses were restricted to European-ancestry samples, given the limited GWAS summary statistics for other populations. Future research should prioritize large-scale GWASs of more diverse samples in order to determine whether the associations of MDD with medical conditions identified here extend to other racial and ethnic groups.

Our findings have several implications. First, they suggest that prevention and treatment of major depressive disorder might help to reduce the morbidity and mortality stemming from a range of associated medical conditions. Second, because individuals with MDD are at greater risk for a broad set of medical conditions, patients with MDD may benefit from integrated care that incorporates medical-disease screening and prevention^68^. Third, our results support the measurement of physical-health outcomes in clinical trials of treatments for major depressive disorder. Such trials could further clarify the potential benefits of MDD treatment for lifespan and healthspan. Lastly, more information is needed concerning the mechanisms linking MDD with diverse medical conditions, and the extent to which mechanisms are shared across conditions. This will require measurement of potential mechanisms within MDD treatment trials and prospective cohort studies, with attention to both behavioral (e.g., health behaviors) and biological (e.g., inflammation) factors.

In conclusion, using a genetically-informed approach for causal inference, this study provides evidence that major depressive disorder may causally increase risk for a broad range of medical conditions. Prevention and treatment of MDD could benefit not only mental health but also physical health.

## Supplementary information

Supplementary Information

Supplementary Data

## Data Availability

The PGC Phase 3 MDD GWAS dataset (excluding 23andMe and UKB) can be downloaded from https://figshare.com/articles/dataset/GWAS_summary_statistics_for_major_depression_PGC_MDD2025_/27061255. GWAS datasets for the other traits used in this study were obtained from the IEU GWAS database (https://opengwas.io/datasets/).

## Code availability

Mendelian randomization analyses were conducted using the open source R package ‘twoSampleMR’ (v0.6.9). The code created for analyses in this paper is available from the corresponding author on reasonable request.
