# Supplementary material for: Testing bidirectional associations of major depressive disorder with medical conditions: two-sample Mendelian randomization study
Source: Npj Ment Health Res. 2026 Apr 9;5:24. doi: 10.1038/s44184-026-00204-7 (PMC13066394; doi:10.1038/s44184-026-00204-7)
Supplement: Supplementary file 1 — Supplementary Information [file 44184_2026_204_MOESM1_ESM.pdf]

Supplemental Information Directory for:

Testing bidirectional associations of major depressive disorder with medical conditions:
Two-sample Mendelian randomization study

Yu Fang, et al.

Supplementary Figure 1. Two-sample Mendelian randomization tests and decision flow chart.

Supplementary Figure 2. Forest plots of the estimated causal effects of MDD on 182 other traits, organized by phecode group.

Supplementary Figure 3. Scatter plots of Mendelian randomization tests in the direction of MDD causing other traits, for the 29 most strongly-associated traits (FDR-corrected p-value < $1x10^{-10}$).

Supplementary Figure 4. Scatter plots of bidirectional Mendelian randomization tests between MDD and 10 MDD-PRS associated traits, in the direction of MDD causing other traits (AA to AJ) and the direction of other traits causing MDD (BA to BJ).

The accompanying Supplementary Data Excel file includes the following:
Supplementary Data 1. 246 phecode traits from published MDD-PRS EHR PheWAS studies
Supplementary Data 2. Candidate and final keywords for phecode - GWAS dataset matching
Supplementary Data 3. Summary of causal effects of MDD on other traits, by phecode group
Supplementary Data 4. Tests of causal effects of MDD on 182 other traits
Supplementary Data 5. Tests of causal effects of 10 other traits on MDD
Supplementary Data 6. Negative control: Tests of causal effects of height on 183 traits

Supplementary Figures

Supplementary Figure 1. Two-sample Mendelian randomization tests and decision flow chart.

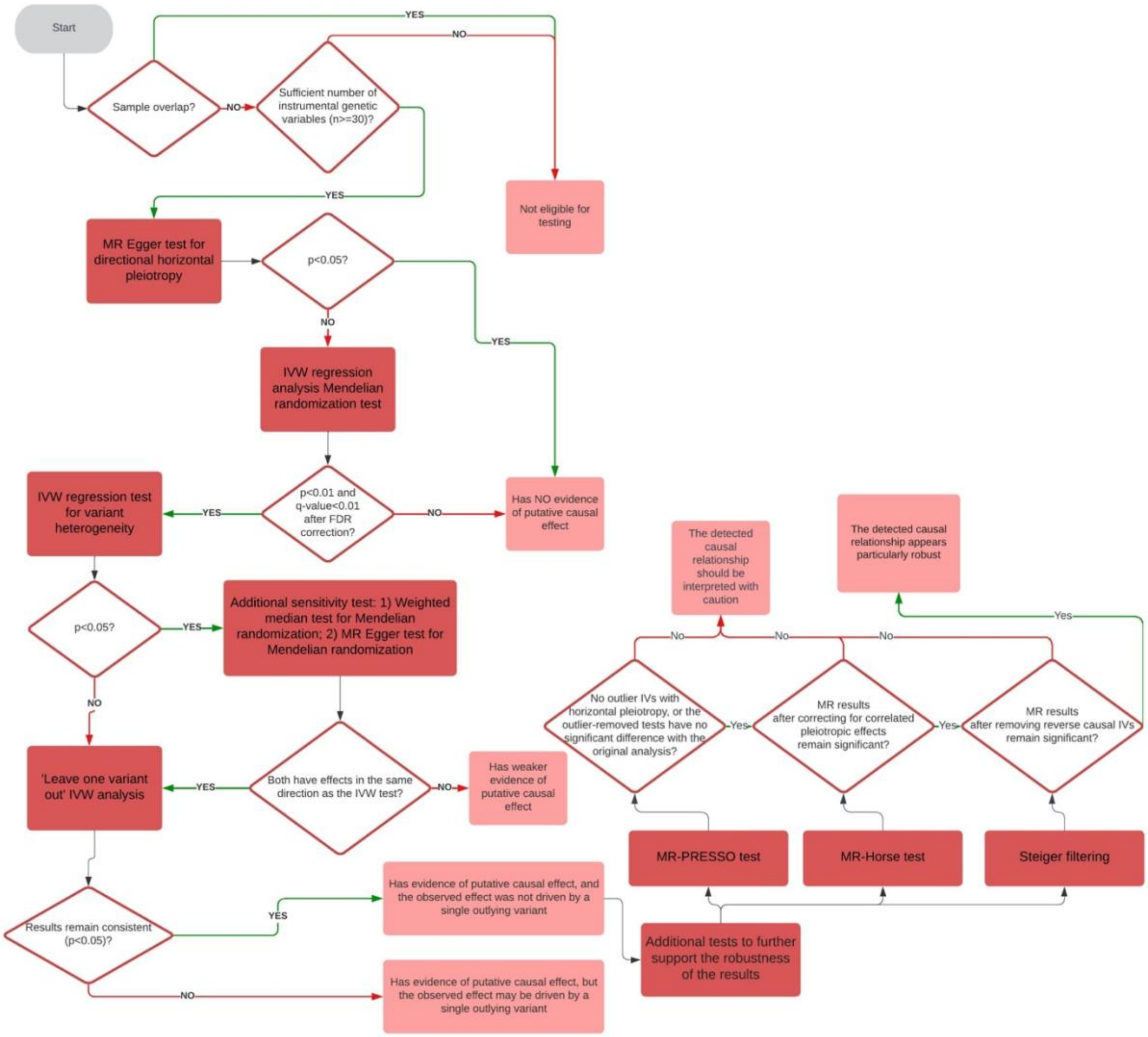

Supplementary Figure 2. Forest plots of the estimated causal effects of MDD on 182 other traits, organized by phecode group.

A.

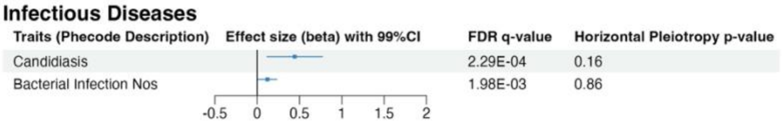

B.

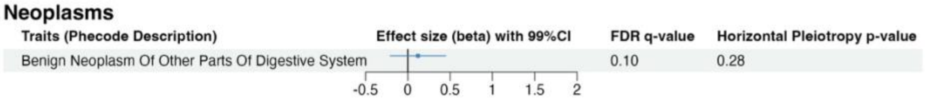

C.

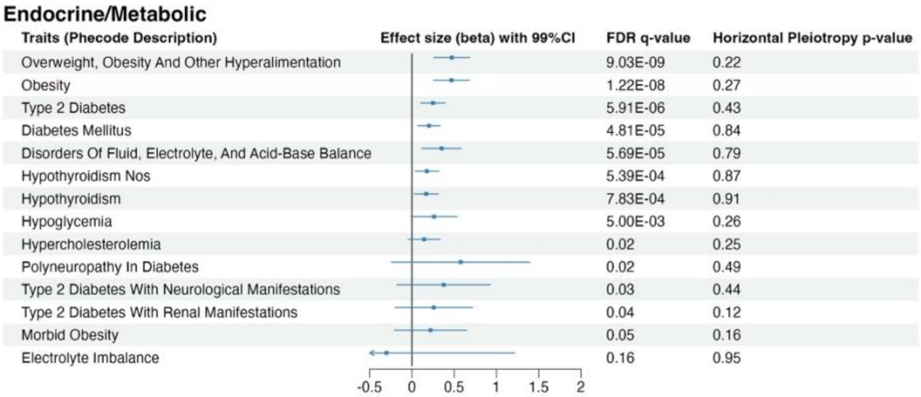

D.

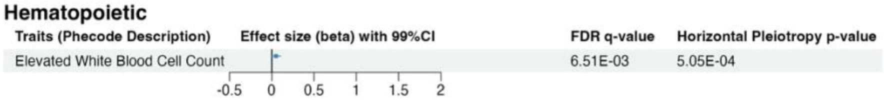

E.

Mental Disorders

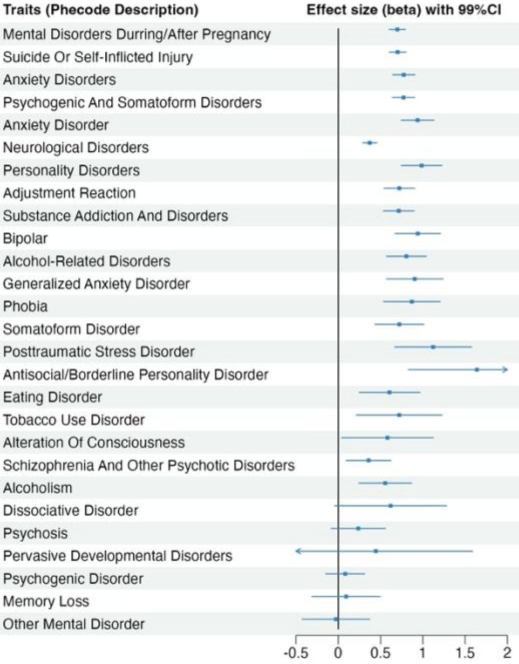

Traits (Phecode Description)	FDR q-value	Horizontal Pleiotropy p-value
Mental Disorders Durring/After Pregnancy	7.15E-76	0.98
Suicide Or Self-Inflicted Injury	2.08E-74	0.95
Anxiety Disorders	4.80E-51	0.41
Psychogenic And Somatoform Disorders	3.01E-50	0.38
Anxiety Disorder	8.70E-35	0.10
Neurological Disorders	7.68E-30	0.26
Personality Disorders	2.50E-25	0.35
Adjustment Reaction	6.98E-24	0.37
Substance Addiction And Disorders	2.05E-23	0.24
Bipolar	3.81E-19	0.12
Alcohol-Related Disorders	3.51E-18	0.35
Generalized Anxiety Disorder	6.10E-12	0.37
Phobia	3.18E-11	0.96
Somatoform Disorder	1.51E-10	0.76
Posttraumatic Stress Disorder	2.47E-10	0.63
Antisocial/Borderline Personality Disorder	1.42E-07	0.33
Eating Disorder	7.92E-06	0.15
Tobacco Use Disorder	1.27E-04	0.86
Alteration Of Consciousness	2.31E-03	0.59
Schizophrenia And Other Psychotic Disorders	2.16E-04	0.07
Alcoholism	2.60E-06	0.04
Dissociative Disorder	5.67E-03	0.60
Psychosis	0.02	2.98E-03
Pervasive Developmental Disorders	0.09	0.54
Psychogenic Disorder	0.10	0.17
Memory Loss	0.15	0.12
Other Mental Disorder	0.22	0.61

F.

Neurological

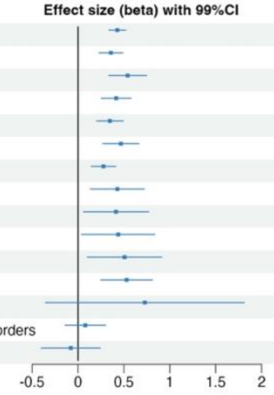

Traits (Phecode Description)	FDR q-value	Horizontal Pleiotropy p-value
Pain	3.95E-32	0.79
Nerve Root And Plexus Disorders	2.19E-12	0.49
Other Headache Syndromes	1.64E-11	0.13
Sleep Apnea	6.04E-11	0.68
Sleep Disorders	8.00E-10	0.92
Migraine	1.13E-09	0.18
Other Peripheral Nerve Disorders	8.26E-08	0.53
Inflammatory And Toxic Neuropathy	9.28E-05	0.93
Abnormal Movement	1.14E-03	0.40
Abnormality Of Gait	1.94E-03	0.25
Insomnia	5.72E-04	0.28
Migrain With Aura	9.04E-07	0.01
Hypersomnia	0.03	0.75
Extrapyramidal Disease And Abnormal Movement Disorders	0.10	0.82
Parkinson's Disease	0.14	0.94

G.

Sense Organs

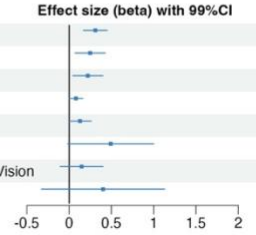

Traits (Phecode Description)	FDR q-value	Horizontal Pleiotropy p-value
Dizziness And Giddiness (Light-Headedness And Vertigo)	1.26E-08	0.39
Visual Disturbances	1.52E-04	0.95
Peripheral Or Central Vertigo	5.72E-04	0.29
Cataract	3.81E-03	0.92
Senile Cataract	4.82E-03	0.75
Otalgia	4.82E-03	0.94
Disorders Of Refraction And Accommodation; Blindness And Low Vision	0.04	0.83
Vertiginous Syndromes And Other Disorders Of Vestibular System	0.05	0.86

H.

Circulatory System

Traits (Phecode Description)	FDR q-value	Horizontal Pleiotropy p-value
Nonspecific Chest Pain	4.22E-27	0.91
Unstable Angina (Intermediate Coronary Syndrome)	2.58E-09	0.92
Angina Pectoris	6.06E-07	0.13
Transient Cerebral Ischemia	9.67E-07	0.57
Heart Failure Nos	2.87E-05	0.76
Myocardial Infarction	3.00E-05	0.19
Symptoms Involving Cardiovascular System	2.05E-04	0.71
Peripheral Vascular Disease	2.42E-03	0.67
Ischemic Heart Disease	2.56E-06	0.04
Essential Hypertension	8.18E-06	6.89E-04
Hypertension	1.31E-04	1.31E-03
Coronary Atherosclerosis	1.48E-04	0.04
Cerebrovascular Disease	2.69E-03	0.20
Occlusion Of Cerebral Arteries	5.14E-03	0.34
Hypertensive Heart And/Or Renal Disease	0.01	0.06
Arrhythmia (Cardiac) Nos	0.02	0.27
Circulatory Disease Nec	0.02	0.85
Other Specified Peripheral Vascular Diseases	0.03	0.68
Hypotension	0.04	0.73
Atherosclerosis	0.04	0.91
Chronic Pulmonary Heart Disease	0.08	0.53
Other Disorders Of Circulatory System	0.09	0.31
Cerebral Ischemia	0.12	0.23
Hypertensive Chronic Kidney Disease	0.20	0.07
Occlusion And Stenosis Of Precerebral Arteries	0.23	0.49
Cardiac Conduction Disorders	0.24	0.41

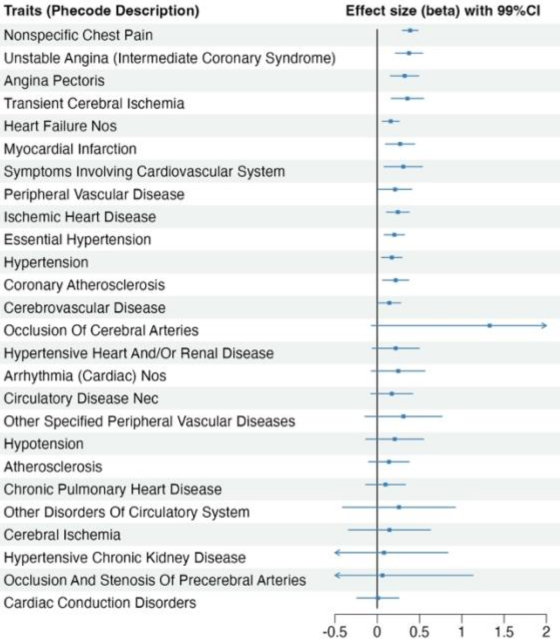

I.

Respiratory

Traits (Phecode Description)	FDR q-value	Horizontal Pleiotropy p-value
Chronic Bronchitis	3.51E-18	0.36
Respiratory Abnormalities	3.80E-18	0.45
Chronic Airway Obstruction	1.41E-16	0.13
Asthma	2.85E-11	0.32
Bronchitis	2.24E-10	0.94
Other Upper Respiratory Disease	1.32E-09	0.19
Acute Upper Respiratory Infections Of Multiple Or Unspecified Sites	6.44E-07	0.36
Pneumonia	8.35E-07	0.27
Chronic Sinusitis	1.66E-06	0.61
Chronic Pharyngitis And Nasopharyngitis	9.81E-05	0.37
Obstructive Chronic Bronchitis	1.47E-06	0.22
Cough	4.92E-03	0.40
Other Symptoms Of Respiratory System	0.02	0.60
Other Dyspnea	0.03	0.35
Allergic Rhinitis	0.07	1.00
Emphysema	0.11	0.19

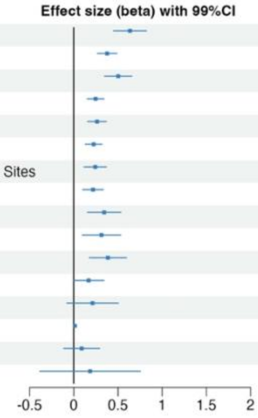

J.

Digestive

Traits (Phecode Description)	FDR q-value	Horizontal Pleiotropy p-value
Gerd	5.88E-82	0.28
Symptoms Involving Digestive System	2.17E-28	0.44
Irritable Bowel Syndrome	6.86E-11	0.38
Arthralgia/Ankylosis Of Temporomandibular Joint	1.64E-10	0.12
Functional Digestive Disorders	4.02E-10	0.51
Dysphagia	1.65E-08	0.72
Diaphragmatic Hernia	3.97E-08	0.39
Noninfectious Gastroenteritis	1.51E-06	0.13
Other Specified Gastritis	8.16E-06	0.65
Heartburn	1.28E-05	0.64
Dyspepsia And Other Specified Disorders Of Function Of Stomach	5.13E-05	0.79
Other Diseases Of The Teeth And Supporting Structures	1.22E-04	0.40
Gingival And Periodontal Diseases	1.03E-03	0.09
Other Chronic Nonalcoholic Liver Disease	1.30E-03	0.13
Dental Caries	1.98E-04	0.08
Diseases Of The Jaws	3.42E-03	0.54
Gastrointestinal Hemorrhage	8.81E-03	0.84
Esophagitis, Gerd And Related Diseases	0.01	0.52
Other Disorders Of Stomach And Duodenum	0.02	0.96
Diseases Of Esophagus	0.02	0.75
Acute Gastritis	0.02	0.74
Diseases Of Hard Tissues Of Teeth	0.02	0.60
Chronic Liver Disease And Cirrhosis	0.03	0.22
Peptic Ulcer (Excl. Esophageal)	0.17	0.63
Disorders Of Esophageal Motility	0.19	0.96
Abdominal Hernia	0.19	0.89
Intestinal Malabsorption (Non-Celiac)	0.20	0.44

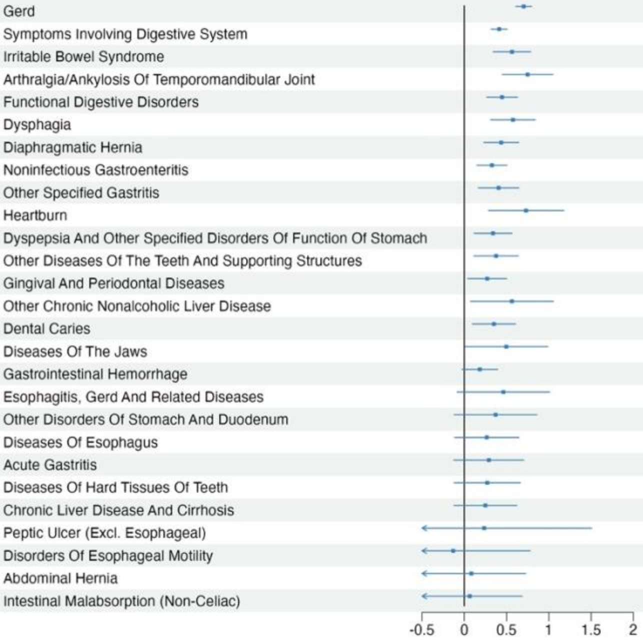

K.

Genitourinary

Traits (Phecode Description)	FDR q-value	Horizontal Pleiotropy p-value
Irregular Menstrual Cycle/Bleeding	6.77E-13	0.92
Urinary Tract Infection	8.03E-12	0.62
Other Symptoms/Disorders Or The Urinary System	2.16E-10	0.24
Ovarian Cyst	9.93E-10	0.84
Cystitis And Urethritis	2.94E-07	0.21
Frequency Of Urination And Polyuria	2.66E-06	0.27
Endometriosis	7.13E-06	0.90
Dysuria	6.12E-05	0.69
Calculus Of Kidney	3.22E-04	0.48
Hematuria	8.18E-04	0.93
Chronic Renal Failure [Ckd]	1.72E-03	0.44
Hyperplasia Of Prostate	2.64E-03	0.42
Urinary Incontinence	4.96E-03	0.74
Acute Renal Failure	0.03	0.62
Renal Failure	0.08	0.26
Urinary Calculus	0.25	0.96

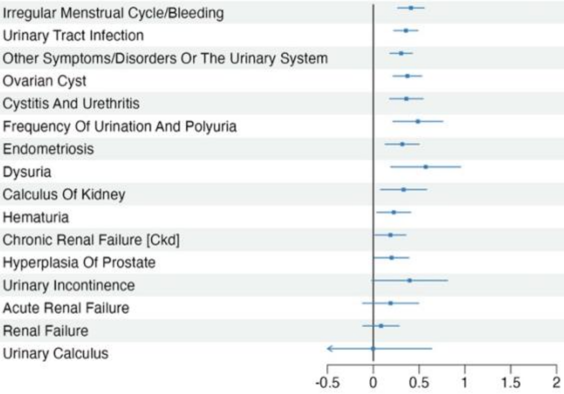

L.

Dermatologic

Traits (Phecode Description)	FDR q-value	Horizontal Pleiotropy p-value
Superficial Cellulitis And Abscess	3.72E-07	0.68
Disturbance Of Skin Sensation	5.95E-06	0.68
Chronic Ulcer Of Skin	0.25	0.78

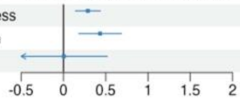

M.

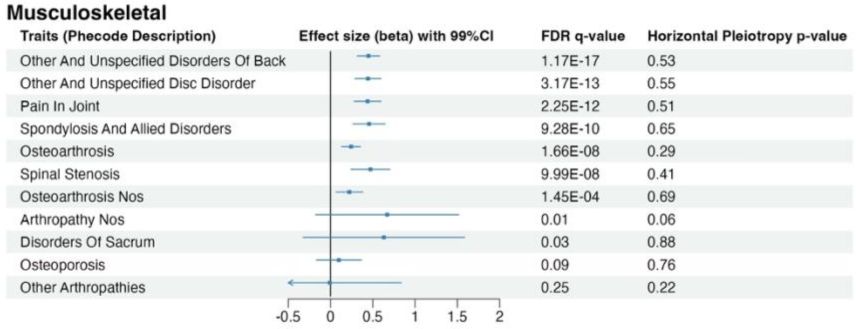

N.

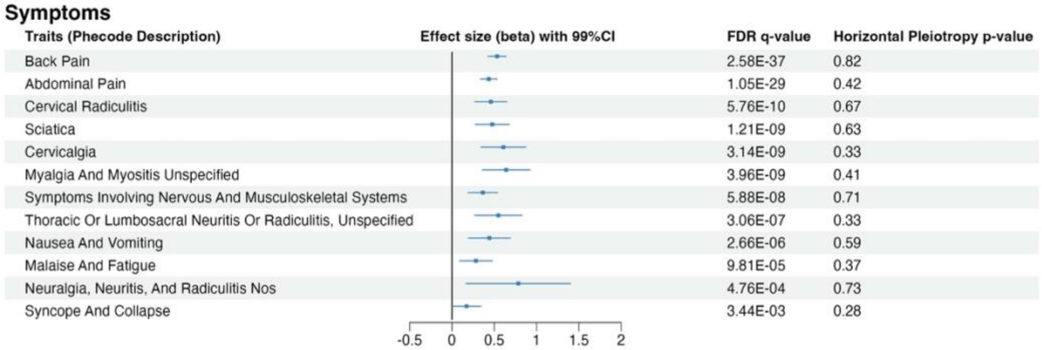

O.

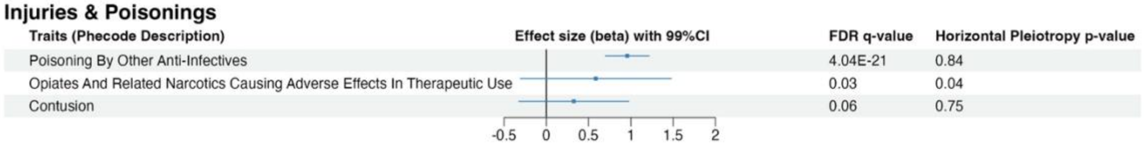

A. Infectious Diseases. B. Neoplasms. C. Endocrine/Metabolic. D. Hematopoietic. E. Mental Disorders. F. Neurological. G. Sense Organs. H. Circulatory System. I. Respiratory. J. Digestive. K. Genitourinary. L. Dermatologic. M. Musculoskeletal. N. Symptoms. O. Injuries & Poisonings. Causal-effect estimates are presented as beta coefficients with 99% confidence intervals. All traits are binary, with the exception of "elevated white blood cell count" (continuous). Odds ratios for associations with binary traits are shown in ***Supplementary Data 4****.*

Supplementary Figure 3. Scatter plots of Mendelian randomization tests in the direction of MDD causing other traits, for the 29 most strongly-associated traits (FDR-corrected p-value < $1x10^{-10}$).

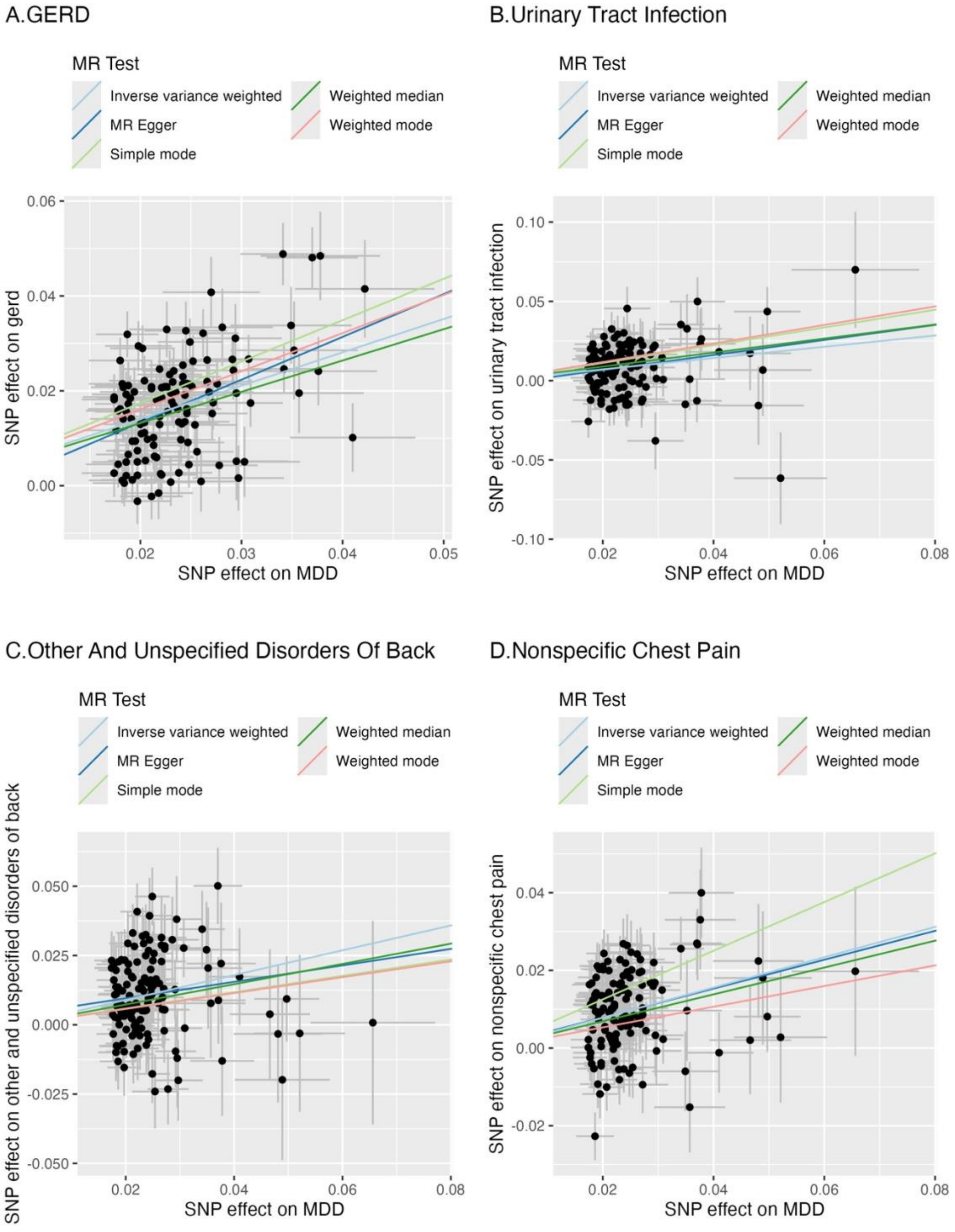

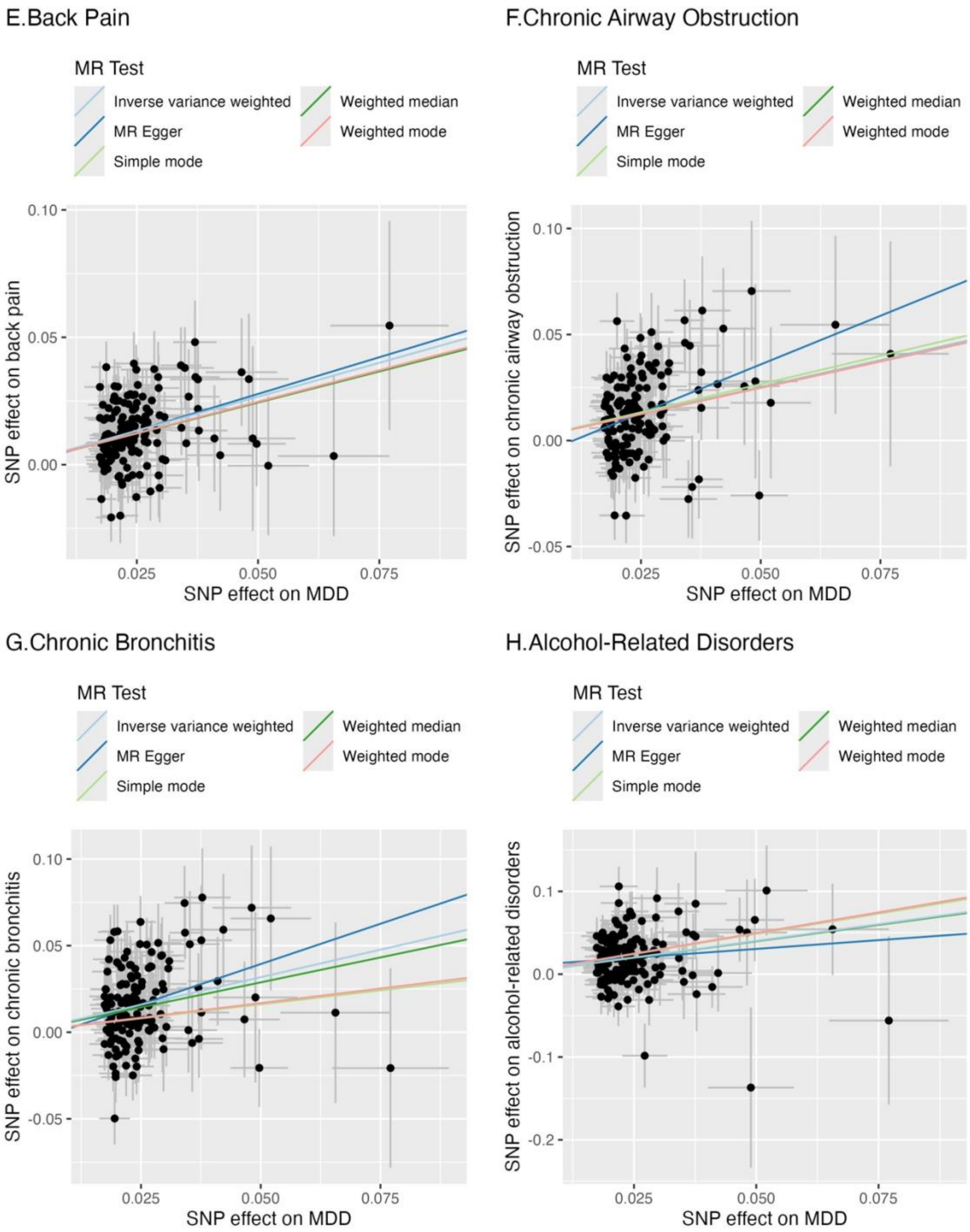

E.Back Pain
MR Test
Inverse variance weighted
MR Egger
Simple mode
Weighted median
Weighted mode
SNP effect on back pain
SNP effect on MDD
F.Chronic Airway Obstruction
MR Test
Inverse variance weighted
MR Egger
Simple mode
Weighted median
Weighted mode
SNP effect on chronic airway obstruction
SNP effect on MDD
G.Chronic Bronchitis
MR Test
Inverse variance weighted
MR Egger
Simple mode
Weighted median
Weighted mode
SNP effect on chronic bronchitis
SNP effect on MDD
H.Alcohol-Related Disorders
MR Test
Inverse variance weighted
MR Egger
Simple mode
Weighted median
Weighted mode
SNP effect on alcohol-related disorders
SNP effect on MDD

I.Anxiety Disorder

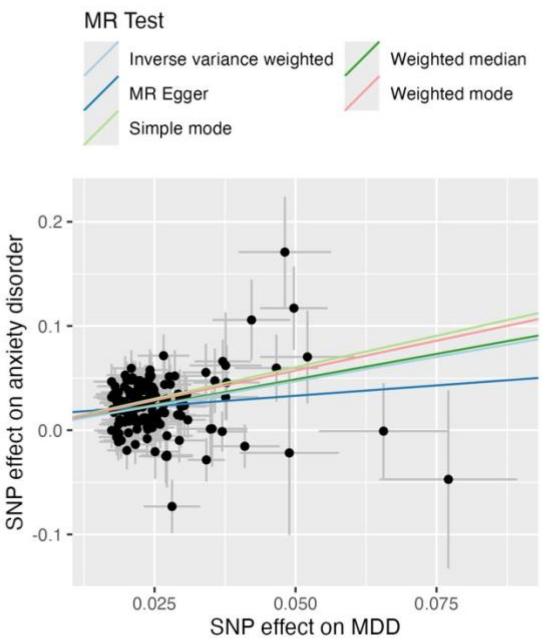

J.Bipolar

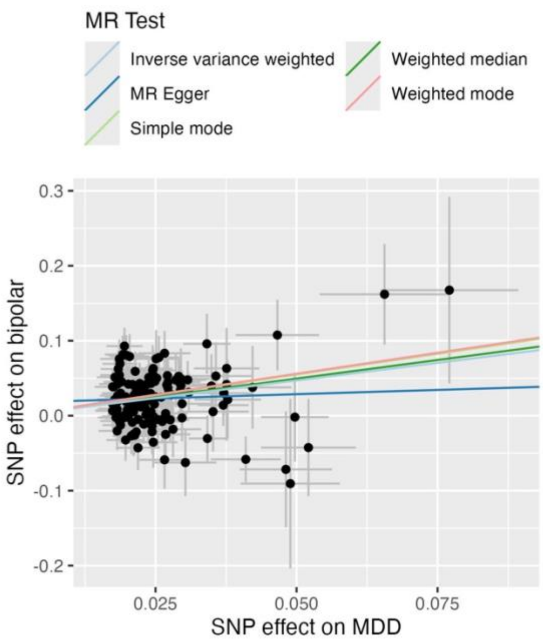

K.Generalized Anxiety Disorder

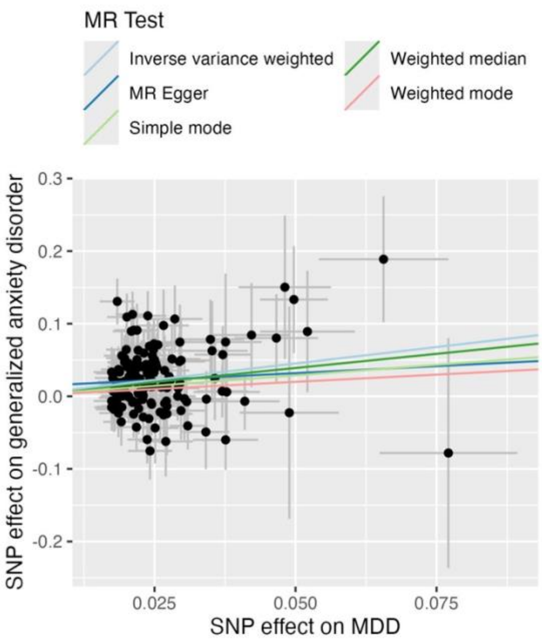

L.Psychogenic And Somatoform Disorders

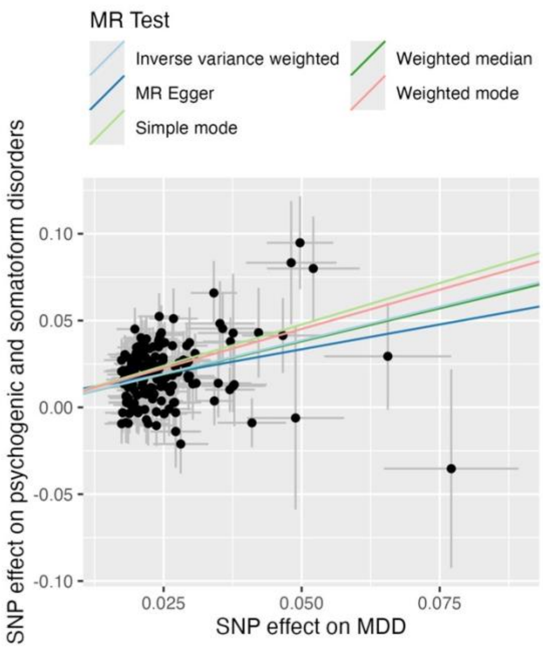

M.Adjustment Reaction

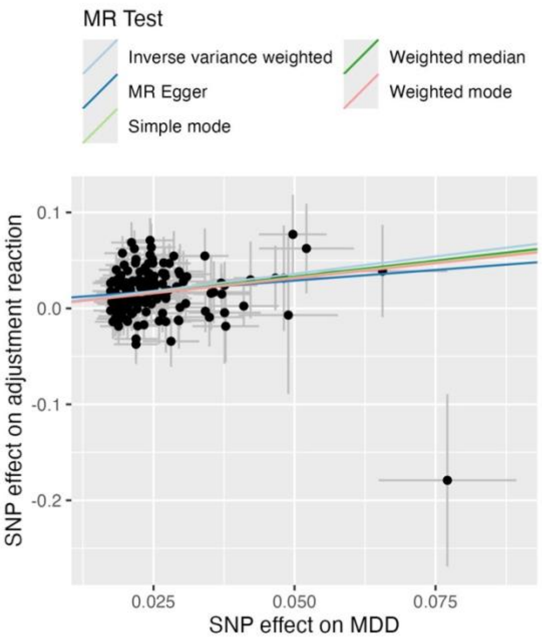

N.Other Headache Syndromes

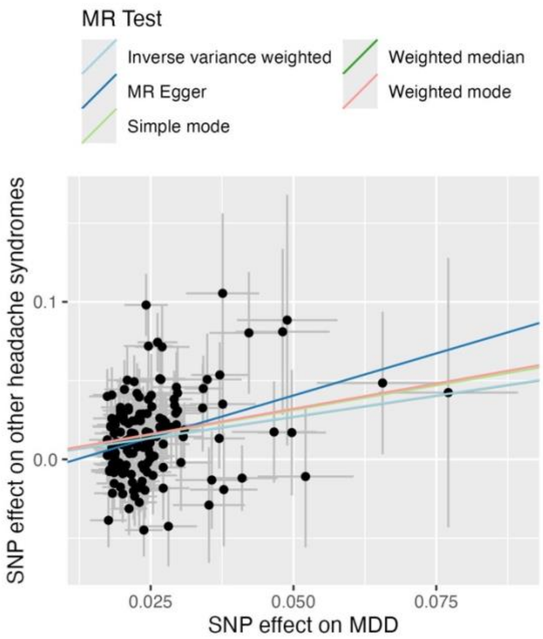

O.Nerve Root And Plexus Disorders

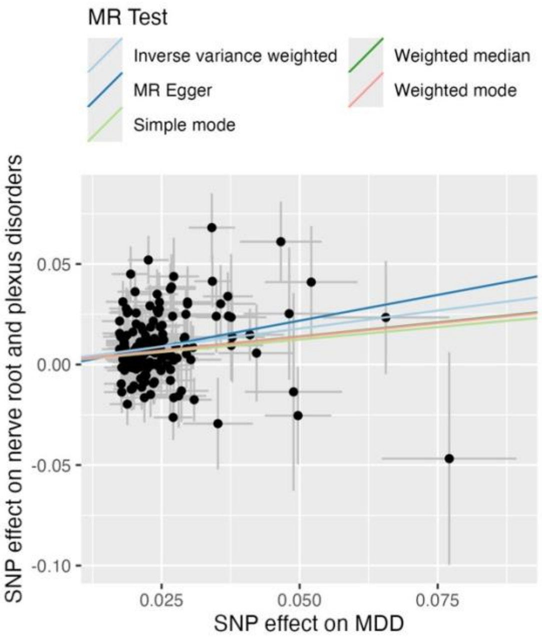

P.Neurological Disorders

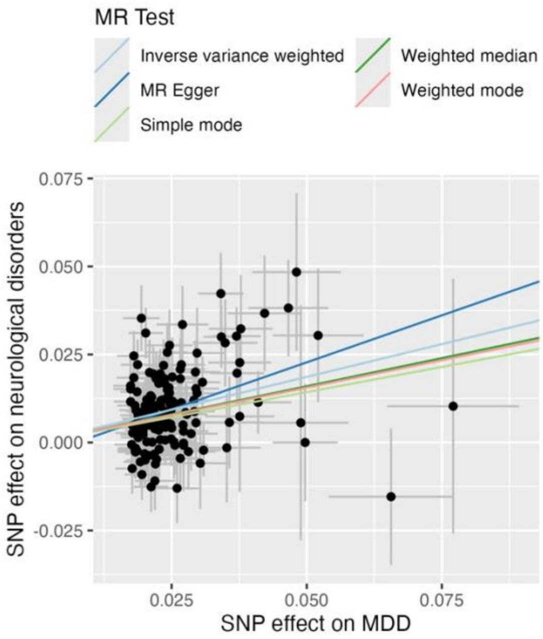

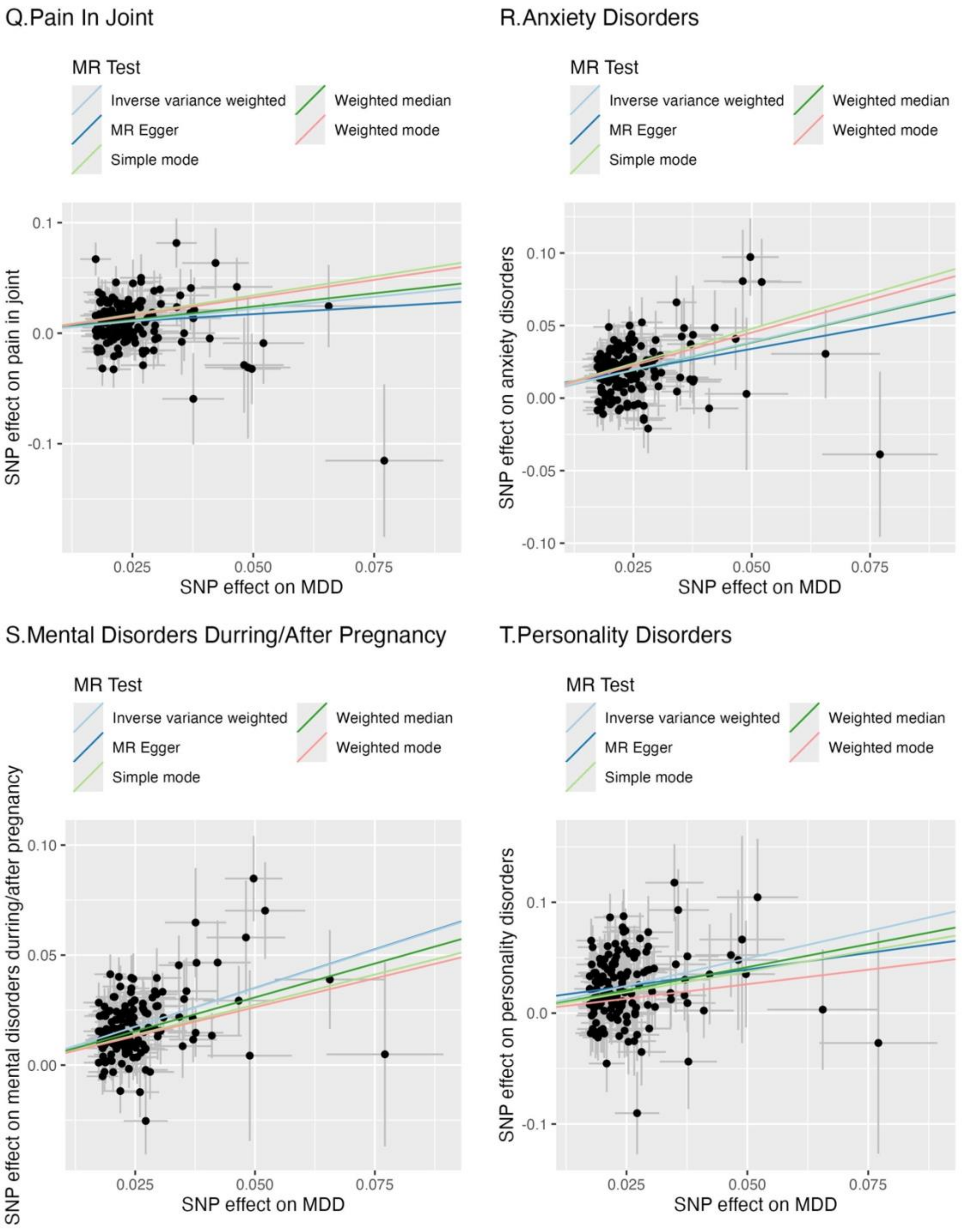
Q.Pain In Joint
R.Anxiety Disorders
S.Mental Disorders Durring/After Pregnancy
T.Personality Disorders
MR Test
Inverse variance weighted
MR Egger
Simple mode
Weighted median
Weighted mode
SNP effect on pain in joint
SNP effect on anxiety disorders
SNP effect on mental disorders durring/after pregnancy
SNP effect on personality disorders
SNP effect on MDD
0.025
0.050
0.075

U.Substance Addiction And Disorders

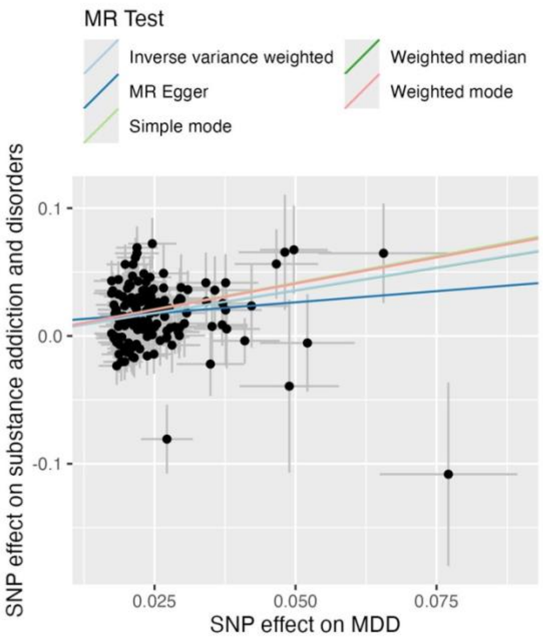

V.Other And Unspecified Disc Disorder

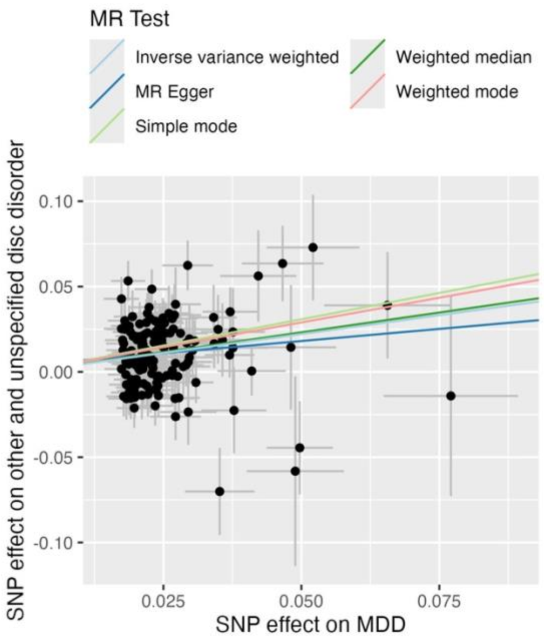

W.Irregular Menstrual Cycle/Bleeding

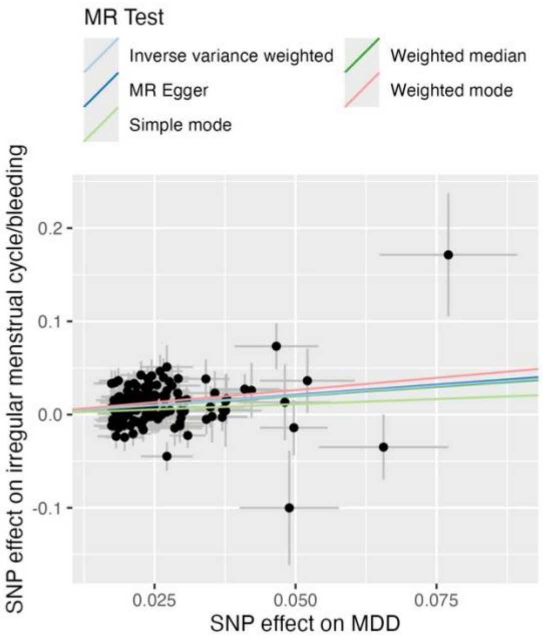

X.Pain

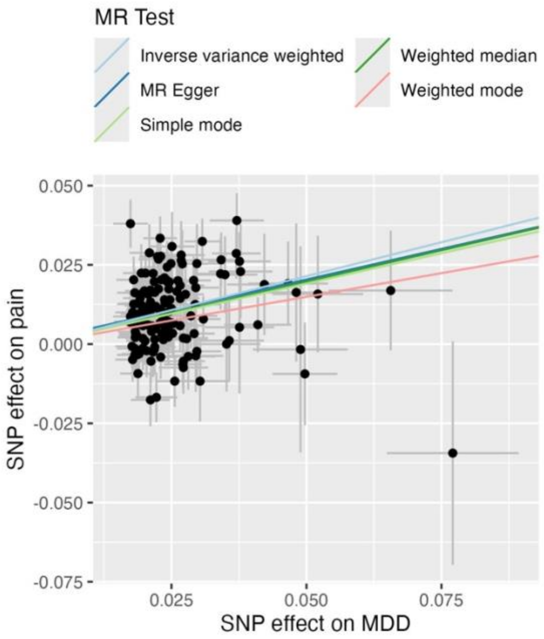

Y.Abdominal Pain

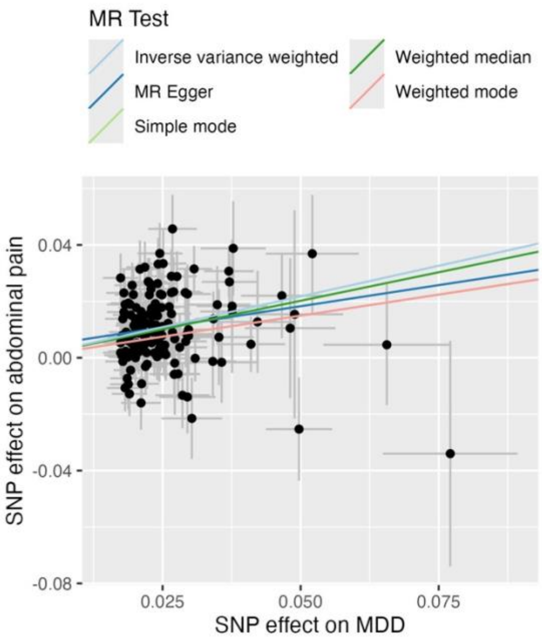

Z.Respiratory Abnormalities

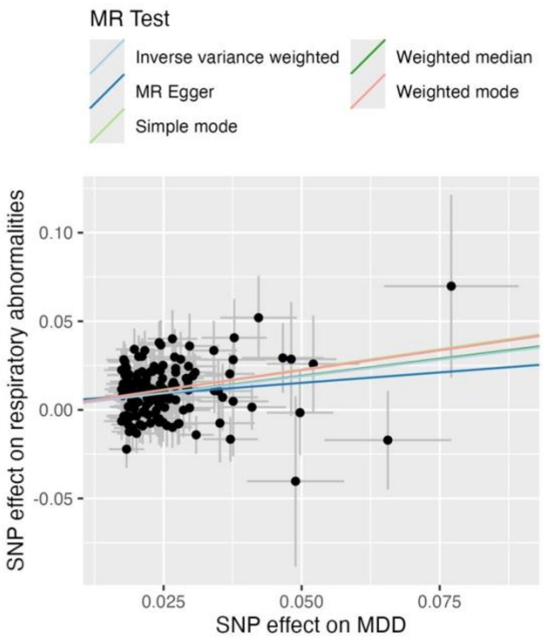

AA.Symptoms Involving Digestive System

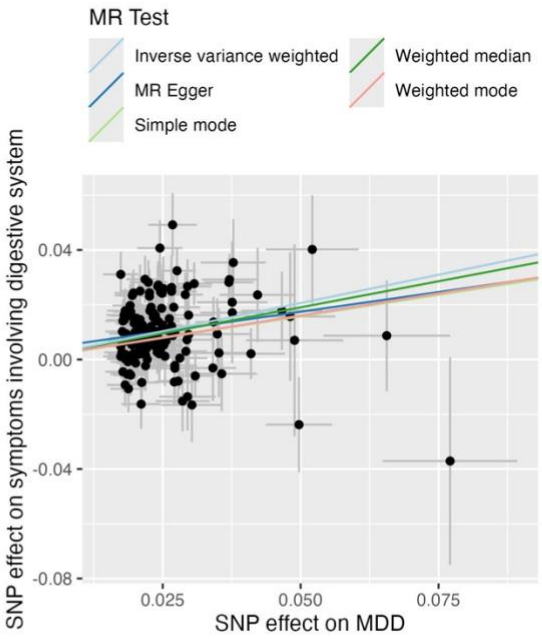

AB.Poisoning By Other Anti-Infectives

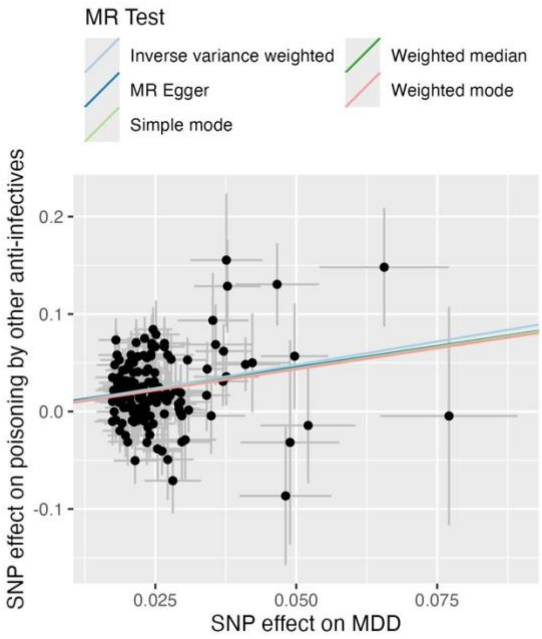

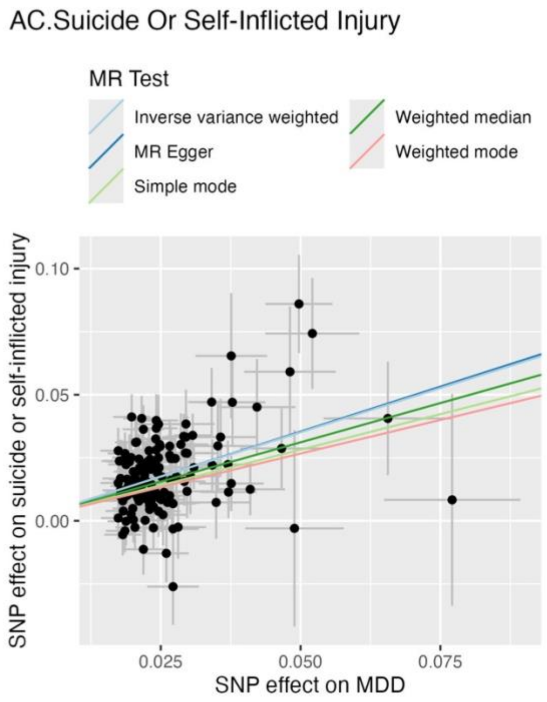

A. GERD. B. Urinary Tract Infection. C. Other and Unspecified Disorders of Back. D. Nonspecific Chest Pain. E. Back Pain. F. Chronic Airway Obstruction. G. Chronic Bronchitis. H. Alcohol-Related Disorders. I. Anxiety Disorder. J. Bipolar. K. Generalized Anxiety Disorder. L. Psychogenic and Somatoform Disorders. M. Adjustment Reaction. N. Other Headache Syndromes. O. Nerve Root and Plexus Disorders. P. Neurological Disorders. Q. Pain in Joint. R. Anxiety Disorders. S. Mental Disorders during/after Pregnancy. T. Personality Disorders. U. Substance Addiction and Disorders. V. Other and Unspecified Disc Disorder. W. Irregular Menstrual Cycle/Bleeding. X. Pain. Y. Abdominal Pain. Z. Respiratory Abnormalities. AA. Symptoms Involving Digestive System. AB. Poisoning by other Anti-infectives. AC. Suicide or Self-inflicted Injury. The slope of each line corresponds to the estimated MR effect per method.

Supplementary Figure 4. Scatter plots of bidirectional Mendelian randomization tests between MDD and 10 MDD-PRS associated traits, in the direction of MDD causing other traits (**AA to AJ**) and the direction of other traits causing MDD (**BA to BJ**).

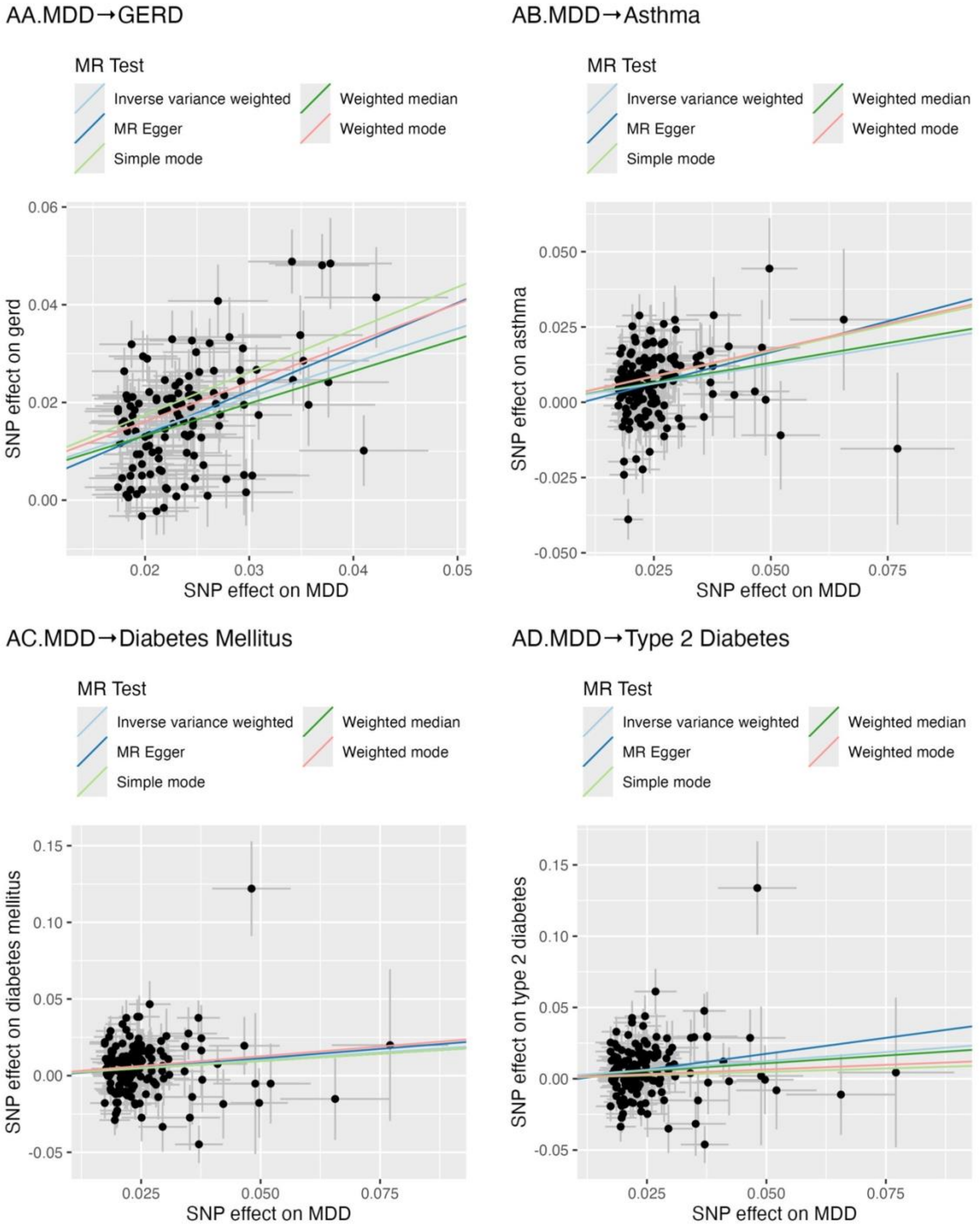

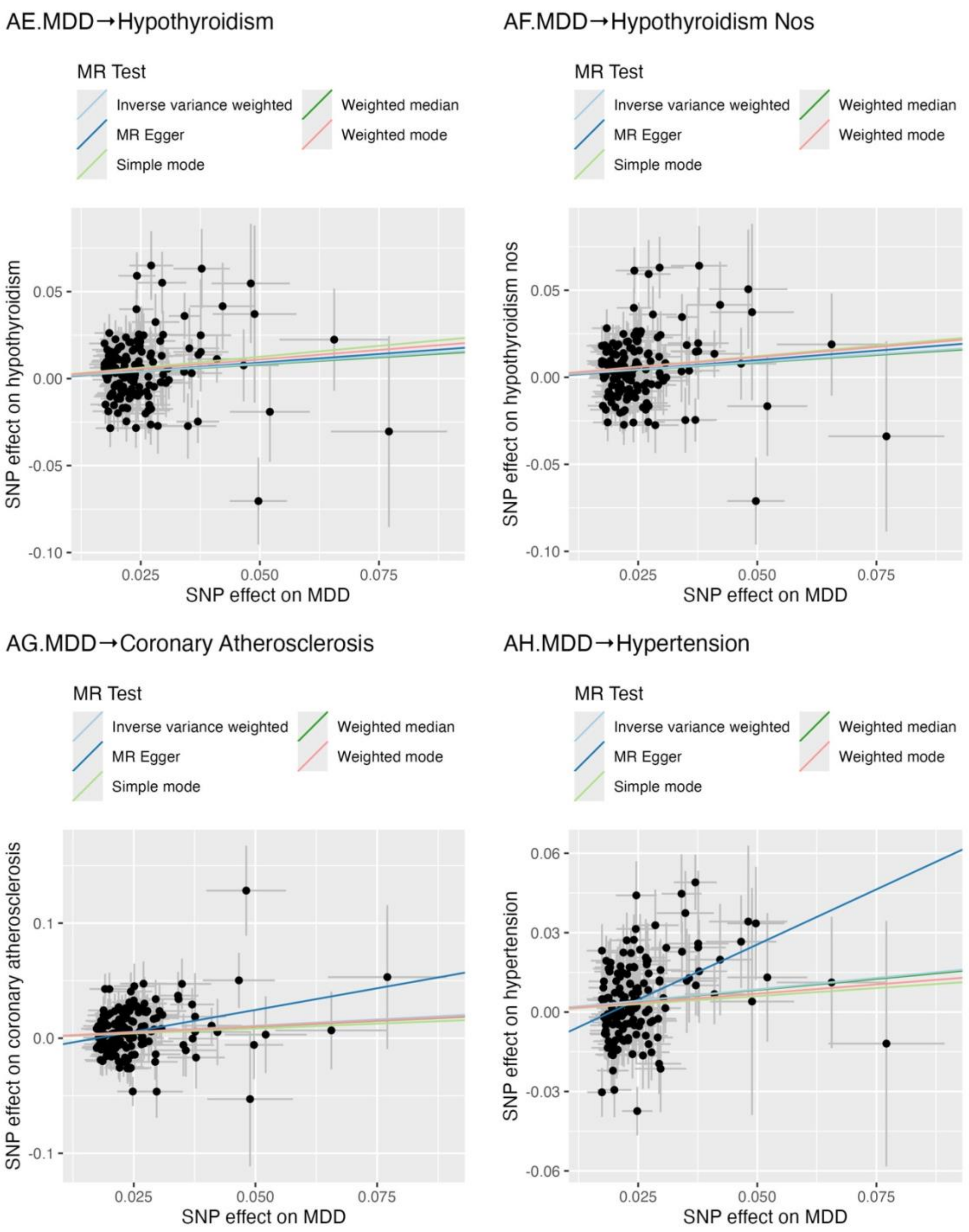
AE.MDD→Hypothyroidism
MR Test
Inverse variance weighted
MR Egger
Simple mode
Weighted median
Weighted mode
SNP effect on hypothyroidism
SNP effect on MDD
AF.MDD→Hypothyroidism Nos
SNP effect on hypothyroidism nos
AG.MDD→Coronary Atherosclerosis
SNP effect on coronary atherosclerosis
AH.MDD→Hypertension
SNP effect on hypertension

AI.MDD→Essential Hypertension

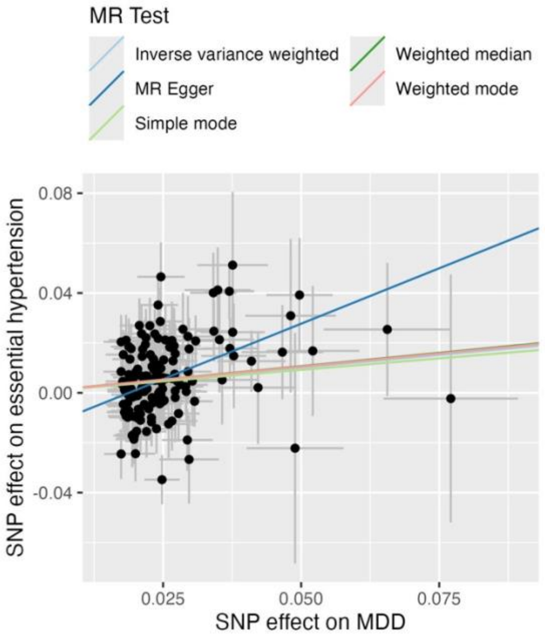

AJ.MDD→Elevated White Blood Cell Count

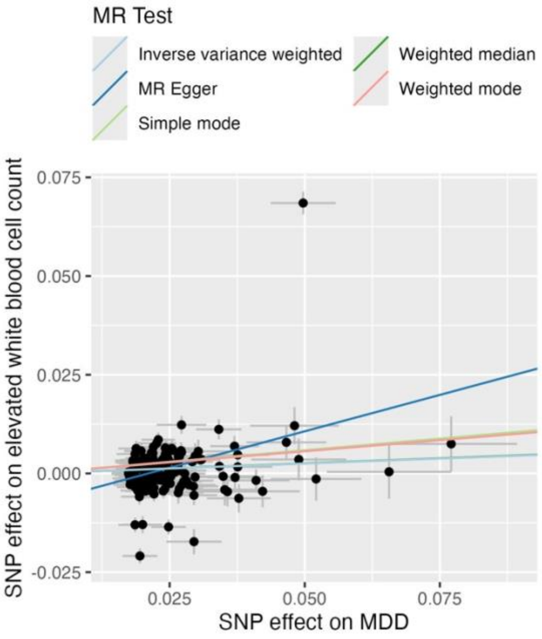

BA.GERD→MDD

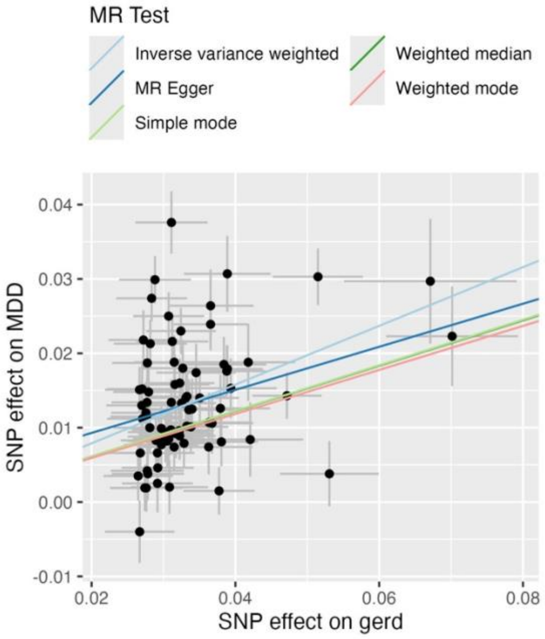

BB.Asthma→MDD

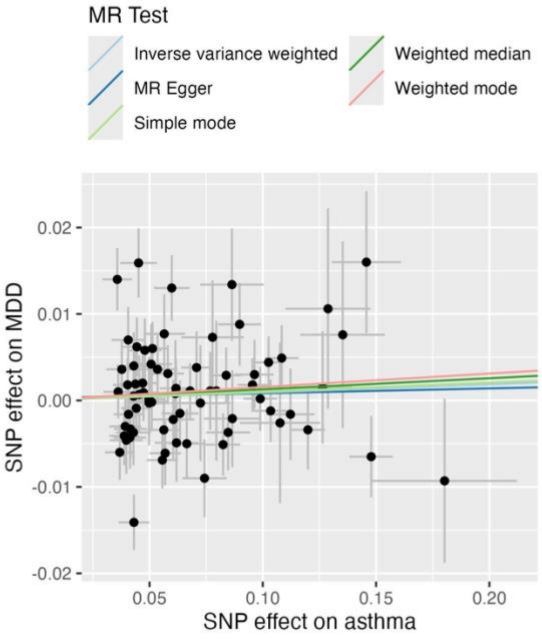

BC.Diabetes Mellitus→MDD

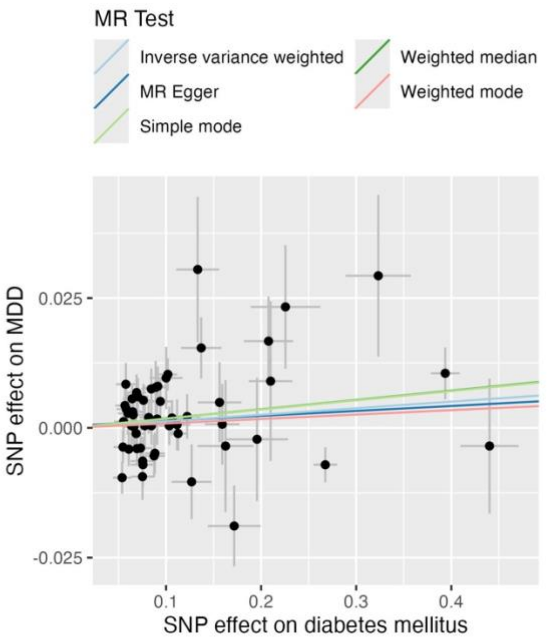

BD.Type 2 Diabetes→MDD

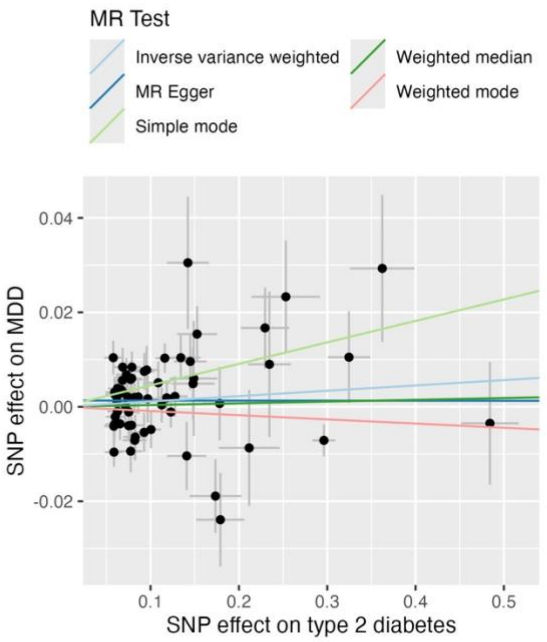

BE.Hypothyroidism→MDD

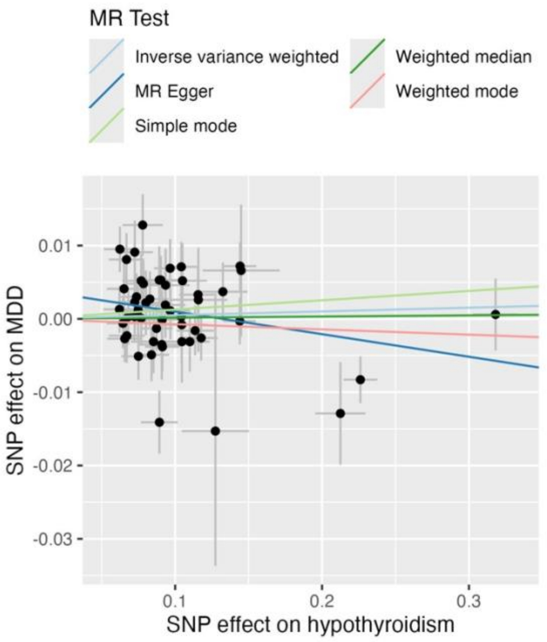

BF.Hypothyroidism Nos→MDD

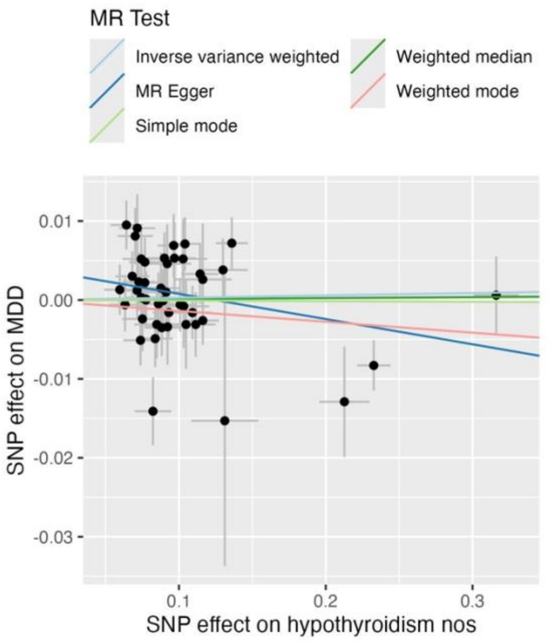

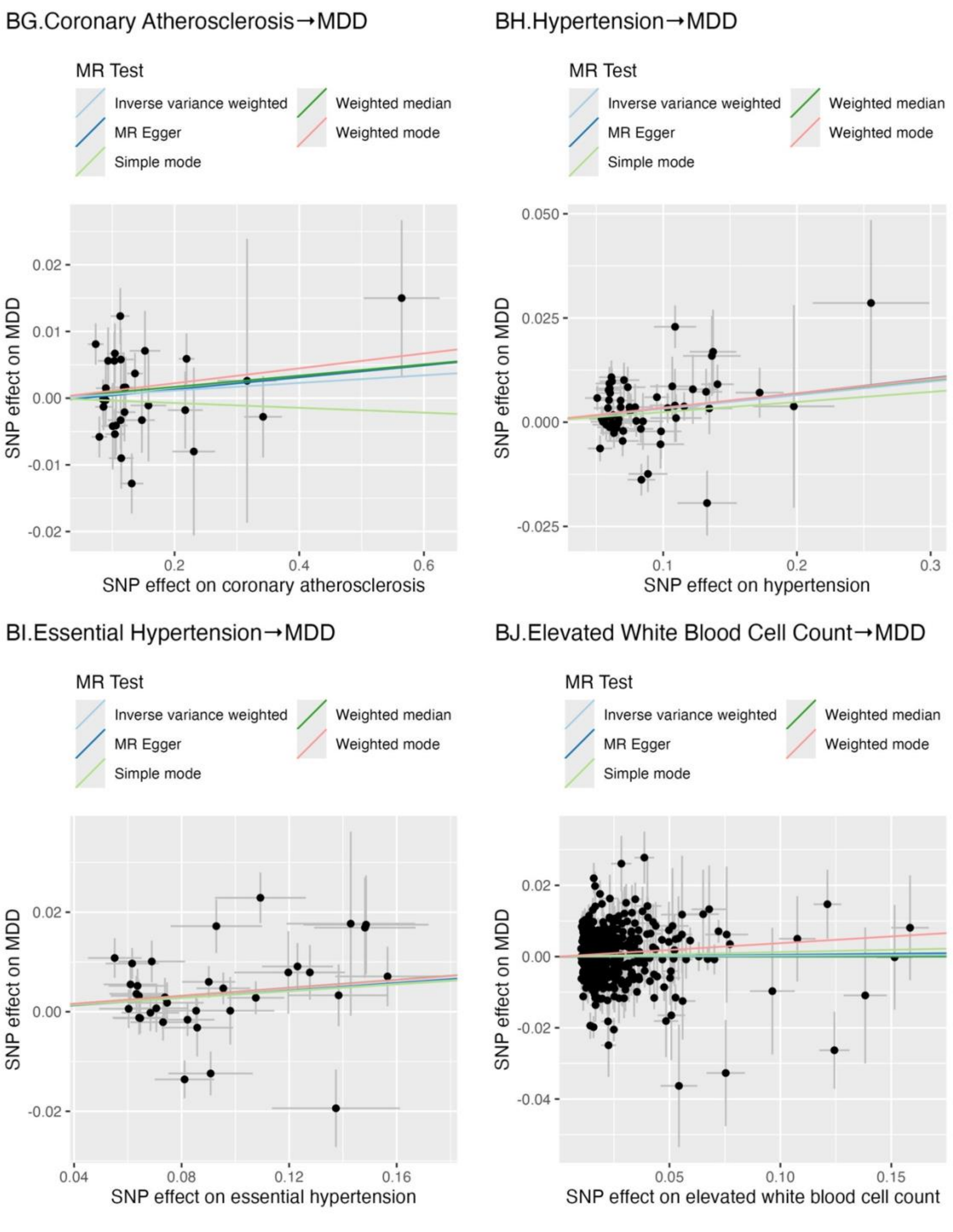

AA. MDD→GERD. AB. MDD→Asthma. AC. MDD→Diabetes Mellitus. AD. MDD→Type 2 Diabetes. AE. MDD→Hypothyroidism. AF. MDD→Hypothyroidism NOS. AG. MDD→Coronary Atherosclerosis. AH. MDD→Hypertension. AI. MDD→Essential Hypertension. AJ. MDD→Elevated White Blood Cell Count. BA. GERD→MDD. BB. Asthma→MDD. BC. Diabetes Mellitus→MDD. BD. Type 2 Diabetes→MDD. BE. Hypothyroidism→MDD. BF. Hypothyroidism NOS→MDD. BG. Coronary

Atherosclerosis→MDD. BH. Hypertension→MDD. BI. Essential Hypertension→MDD. BJ. Elevated White Blood Cell Count→MDD. The slope of each line corresponds to the estimated MR effect per method.
